# Prematurity blunts protein synthesis in skeletal muscle independently of body weight in neonatal pigs

**DOI:** 10.1038/s41390-022-02456-3

**Published:** 2023-01-10

**Authors:** Marko Rudar, Jane K. Naberhuis, Agus Suryawan, Hanh V. Nguyen, Marta L. Fiorotto, Teresa A. Davis

**Affiliations:** 1 Department of Animal Sciences, Auburn University, Auburn, AL USA; 2 United States Department of Agriculture/Agricultural Research Service Children’s Nutrition Research Center, Department of Pediatrics, Baylor College of Medicine, Houston, TX USA

## Abstract

**Background:**

Postnatal growth failure in premature infants is associated with reduced lean mass accretion. Prematurity impairs the feeding-induced stimulation of translation initiation and protein synthesis in the skeletal muscle of neonatal pigs. The objective was to determine whether body weight independently contributes to the blunted postprandial protein synthesis.

**Methods:**

Preterm and term pigs that were either fasted or fed were stratified into quartiles according to birth weight to yield preterm and term groups of similar body weight; first and second quartiles of preterm pigs and third and fourth quartiles of term pigs were compared (preterm-fasted, *n* = 23; preterm-fed, *n* = 25; term-fasted, *n* = 21; term-fed, *n* = 21). Protein synthesis rates and mechanistic target of rapamycin complex 1 (mTORC1) activation in skeletal muscle were determined.

**Results:**

Relative body weight gain was lower in preterm compared to term pigs. Prematurity attenuated the feeding-induced increase in mTORC1 activation in longissimus dorsi and gastrocnemius muscles (*P* < 0.05). Protein synthesis in gastrocnemius (*P* < 0.01), but not in longissimus dorsi muscle, was blunted by preterm birth.

**Conclusion:**

A lower capacity of skeletal muscle to respond adequately to feeding may contribute to reduced body weight gain and lean mass accretion in preterm infants.

**Impact:**

- This study has shown that the feeding-induced increase in protein synthesis of skeletal and cardiac muscle is blunted in neonatal pigs born preterm compared to pigs born at term independently of birth weight.
- These findings support the notion that preterm birth, and not low birth weight, impairs the capacity of skeletal and cardiac muscle to upregulate mechanistic target of rapamycin-dependent anabolic signaling pathways and protein synthesis in response to the postprandial increase in insulin and amino acids.
- These observations suggest that a blunted anabolic response to feeding contributes to reduced lean mass accretion and altered body composition in preterm infants.

## Introduction

Nutritional support for premature infants aims to achieve postnatal growth rate and body composition that approximates a normal fetus of the same gestational age.^1^ Infants born premature have less lean mass and more body fat compared to infants born at term at an equivalent age,^2^ which can increase the lifelong risk for metabolic disease.^3–6^ Preterm birth also has been linked to poor neurodevelopmental outcomes,^7,8^ although a direct relationship between postnatal growth rate and cognitive development remains controversial.^9–11^

Growth in early postnatal life is characterized by a rapid increase in skeletal muscle mass,^12,13^ and the accretion of skeletal muscle mass in the neonate is uniquely sensitive to both insulin and amino acids compared to later stages of postnatal life.^14–16^ Insulin and amino acids promote the activation of mechanistic target of rapamycin (mTOR) complex 1 (mTORC1), an integral protein kinase that regulates translation initiation and protein synthesis in skeletal muscle and other tissues.^17^ Previously, we demonstrated that prematurity attenuates the feeding-, insulin-, and amino acid-induced activation of mTORC1 and protein synthesis in skeletal muscle in a neonatal pig model of prematurity.^18,19^ However, it is unclear whether the lower birth weight of preterm compared to term neonates is an independent risk factor for perturbed anabolic signaling and protein synthesis in muscle. In this secondary analysis of previously published data,^18^ we aimed to evaluate the relationship between birth weight, mTORC1 activation, and protein synthesis in skeletal muscle of neonatal pigs born either preterm or at term. We hypothesized that the blunted anabolic response to feeding in preterm pigs compared to term pigs is independent of birth weight.

## Methods

### Animals and housing

The experimental protocol was approved by the Institutional Animal Care and Use Committee of Baylor College of Medicine. The experiment was carried out in compliance with the National Research Council’s *Guide for the Care and Use of Laboratory Animals* as described in Naberhuis et al.^18^

Surgeries were performed under general isoflurane anesthesia and with an aseptic technique. Seven pregnant sows were procured from a commercial swine farm and housed with ad libitum access to a commercial diet (Laboratory Porcine Grower Diet 5084, LabDiet, St. Louis, MO; metabolizable energy, 3160 kcal/kg diet; crude protein, 160 g/kg diet) and water at the USDA/ARS Children’s Nutrition Research Center (Houston, TX) for 1 week before study procedures. Piglets were delivered via Cesarean section at either day 103 of gestation (preterm) or day 112 of gestation (term), resuscitated, surgically fitted with jugular vein and carotid artery catheters, and housed in plexiglass incubators maintained between 29 and 32 °C and on a 12-h light/12-h dark cycle, as described previously.^18^ All pigs received iron dextran (30 mg i.m.; Henry Schein Animal Health, Dublin, OH), meloxicam SR (0.6 mg·kg^–1^ i.m.; ZooPharm, Laramie, WY), and sterile sow plasma (4, 5, and 7 mL·kg^–1^ i.v. at 6, 12, and 24 h, respectively, after birth). Pigs were monitored hourly for the full duration of the study, and complete clinical evaluations (mentation, urination, defecation, vomiting, temperature, gait, respiration, skin color, mucus membrane perfusion, vocalization, and indications of pain or discomfort) were performed every 12 h. Institutional veterinarians were consulted if any parameter was abnormal.

### Study design and nutrition support

Total parenteral nutrition was formulated to meet or exceed nutrient requirements of neonatal pigs^20,21^ and was administered for 4 days, as described previously.^18^ On day 4, all piglets were fasted for 4 h, placed in a sling restraint system, and were either fasted for one additional hour or fed an elemental meal providing one-sixth of daily nutrient requirements by oral gavage, yielding four groups: preterm-fasted (PT-FAST; *n* = 23, 13 males and 10 females); preterm-fed (PT-FED; *n* = 25, 14 males and 11 females); term-fasted (T-FAST; *n* = 21, 11 males and 10 females); and term-fed (T-FED; *n* = 21, 16 males and 5 females). A flooding dose of L-[4-^3^H]-Phe (1.50 mmol Phe·kg^–1^, 0.5 mCi of L-[4-^3^H]-Phe·kg^–1^, American Radiolabeled Chemicals, St. Louis, MO) was injected intravenously into all pigs 30 min before euthanasia. Following euthanasia (60 min after the end of the additional fasting time in the FAST groups or 60 min after feeding in the FED groups; Beuthanasia-D, 0.45 mL·kg^–1^ i.v.; Merck Animal Health, Kenilworth, NJ), longissimus dorsi (LD), gastrocnemius, diaphragm, heart (left ventricle), liver, lung, jejunum, pancreas, kidney, and brain (forebrain) tissues were excised, snap-frozen in liquid nitrogen, and stored at –80 °C until analysis. Body weight was measured daily until euthanasia; relative body weight gain was calculated from the change in body weight from birth to euthanasia.

### Tissue protein synthesis and tissue protein immunoprecipitation and immunoblot analysis

Fractional protein synthesis rates (%/day) were determined by measuring the incorporation of L-[4-^3^H]-Phe into tissue protein as described previously.^18,22^ Immunoprecipitation of eukaryotic initiation factor (eIF)4E·eIF4G in skeletal muscle was performed as described previously.^23^ Immunoprecipitation of insulin receptor substrate (IRS)1·phosphatidylinositiol 3-kinase (PI3K), mTOR·Ras homolog enriched in brain (Rheb), Stress response protein 2 (Sestrin2)·GTPase-activating protein toward Rags 2 (GATOR2), mTOR·Ras-related GTP binding protein (Rag)A, and mTOR·RagC in skeletal muscle were performed as described previously.^18,23^ The phosphorylation and/or total abundance of proteins involved in insulin signaling upstream of mTORC1 (insulin receptor [IR], IRS1, PI3K, Akt, and tuberous sclerosis complex 2 [TSC2]), translation initiation and elongation factor activation downstream of mTORC1 (eIF4E binding protein 1 [4EBP1], ribosomal protein S6 kinase-1 [S6K1], eIF2α, and eukaryotic elongation factor 2 [eEF2]), and protein degradation (Forkhead box O3 [FoxO3], microtubule-associated protein light chain [LC3]-II: total LC3, muscle ring finger protein 1 [MuRF1], and muscle atrophy F-box [atrogin-1]) in skeletal muscle and other tissues were measured by immunoblot analysis. Antibodies were obtained from the following sources: eIF4E (immunoprecipitation of eIF4E·eIF4G; gift of Dr Leonard Jefferson, Pennsylvania State University, College of Medicine, Hershey, PA), eIF4G (catalog no. 07-1800; Millipore Sigma, Burlington, MA), eIF4E (catalog no. 9742; Cell Signaling Technology, Danvers, MA), IRS1 (immunoprecipitation of IRS·P13K; catalog no. 3407, Cell Signaling Technology), pan-phospho-Tyr (IRS1 phosphorylation following immunoprecipitation; catalog no. 9411, Cell Signaling Technology), PI3K (catalog no. 4292, Cell Signaling Technology), Raptor (immunoprecipitation of mTORC1; catalog no. 2280, Cell Signaling Technology), Rheb (catalog no. 13879, Cell Signaling Technology), RagA (catalog no. 4357, Cell Signaling Technology), RagC (catalog no. 5466, Cell Signaling Technology), mTOR (catalog no. 2972, Cell Signaling Technology), Mios (GATOR2 subunit for immunoprecipitation of Sestrin2·GATOR2; catalog no. 13557, Cell Signaling Technology), Sestrin2 (catalog no. 8487, Cell Signaling Technology), phospho-IR Tyr1185 (catalog no. orb5550, Biorbyt LCC, San Francisco, CA), total IR (catalog no. sc-711, Santa Cruz Biotechnology, Dallas, TX), phospho-Akt Ser473 (catalog no. 9271, Cell Signaling Technology), total Akt (catalog no. 9272, Cell Signaling Technology), phospho-TSC2 Thr1462 (catalog no. 3617, Cell Signaling Technology), total TSC2 (catalog no. 4308, Cell Signaling Technology), phospho-4EBP1 Thr70 (catalog no. 9455, Cell Signaling Technology), phospho-S6K1 Thr389 (catalog no. AF8963, R&D Systems, Minneapolis, MN), total S6K1 (catalog no. 14485-1-AP, ProteinTech Group, Rosemont, IL), phospho-eIF2α Ser51 (catalog no. SAB4504388, Millipore Sigma), total eIF2α (catalog no. BS3651, bioWORLD, Dublin, OH), phospho-eEF2 Thr56 (catalog no. 2331, Cell Signaling Technology), total eEF2 (catalog no. 2332, Cell Signaling Technology), phospho-FoxO3 Ser254 (catalog no. 9466, Cell Signaling Technology), total FoxO3 (catalog no. 2497, Cell Signaling Technology), LC3A/B (catalog no. 4108, Cell Signaling Technology), total MuRF1 (catalog no. AF5366, R&D Systems), total atrogin-1 (catalog no. AP2041, ECM Biosciences, Versailles, KY), and total GAPDH (catalog no. 60004-1-Ig, ProteinTech Group). Total protein abundance was normalized to either GAPDH or vinculin. Phosphoprotein abundance was normalized to the abundance of the corresponding native protein; phospho-4EBP1 abundance was normalized to the sum of phosphorylated and non-phosphorylated 4EBP1 analyzed from the same antibody. Additional technical details and antibody dilutions are reported elsewhere.^18,23^

### Statistical analysis

The correlation and generalized linear mixed model procedures of SAS software (version 9.4; SAS Institute, Cary, NC) were used to perform statistical analysis. Within gestational age at birth (GAB; preterm or term), Pearson correlation coefficients were determined between birth weight and relative body weight gain. Within each treatment group, Pearson correlation coefficients were determined between birth weight, skeletal muscle fractional protein synthesis rates, and skeletal muscle signaling protein abundance.

Pigs within each treatment group were subsequently stratified into quartiles for birth weight; the topmost two quartiles for preterm pigs and the lowermost two quartiles for term pigs were selected for analysis (i.e., preterm pigs with the largest birth weight were compared to term pigs with the smallest birth weight; PT-FAST, *n* = 12, 8 male and 4 female; PT-FED, *n* = 13, 9 male and 4 female; T-FAST, *n* = 10, 4 male and 6 female; T-FED, *n* = 10, 6 male and 4 female). All animals from each stratified cohort were included in the analysis. One-factor ANOVA was used to analyze differences in birth weight, final weight, and relative body weight gain between preterm and term pigs. Two-factor ANOVA was used to analyze differences in tissue protein synthesis and signaling protein abundance or activation with GAB and feeding status (STATE; fasted or fed) as the main effects. Sex was included in the initial statistical model as the main effect; however, sex was not significant for any response variable measured and was thus excluded from the final statistical model. Pig was considered the experimental unit; pig and litter were considered random effects. Studentized residuals were analyzed for normality with the Shapiro–Wilk test statistic. Means were compared using Tukey’s post hoc test. Data are presented as least-squares means ± standard error. Differences among groups were considered significant at *P* < 0.05 and a trend at *P* < 0.10.

## Results

**Fig. 1:**
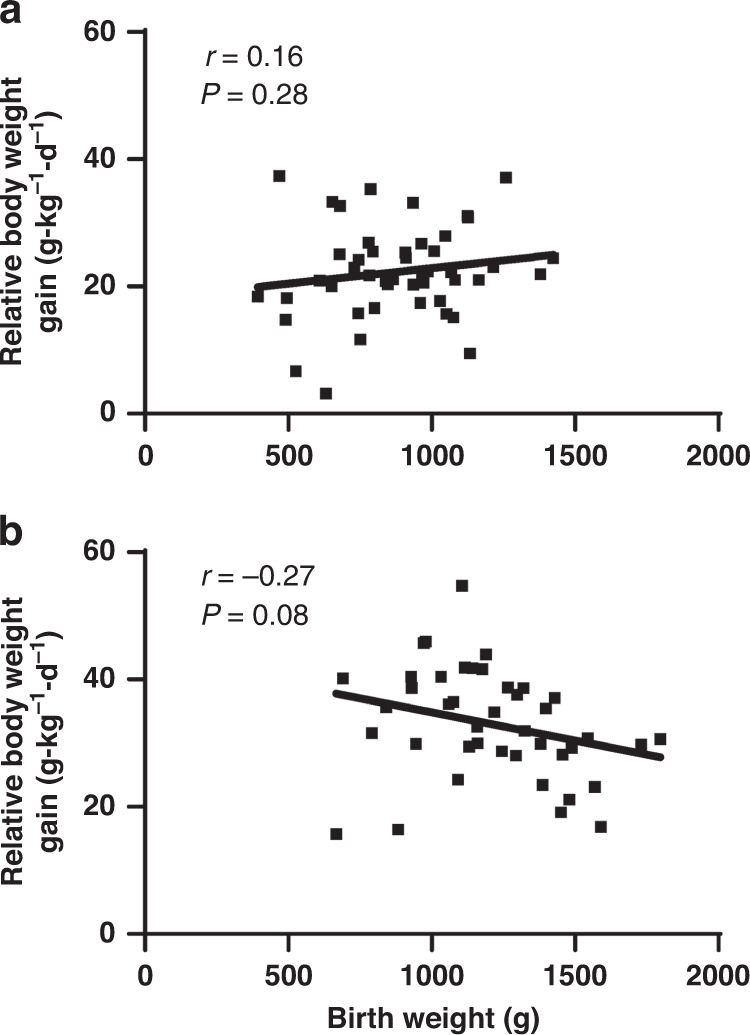
Pearson correlations between birth weight and relative body weight gain in all preterm and term pigs. Preterm pigs (**a**), *n* = 48 (27 male and 21 female); term pigs (**b**), *n* = 42 (26 male and 16 female).

Birth weights ranged from 394 to 1424 g in all preterm and 667 to 1797 g in all term pigs.^18^ Relative postnatal body weight gain was not correlated with birth weight in preterm pigs (*r* = 0.16, *P* = 0.28; Fig. 1a) and tended to be weakly and negatively correlated in term pigs (*r* = –0.27, *P* = 0.08; Fig. 1b). To determine whether body weight independently contributes to the blunted anabolic response to nutrition, preterm and term pigs from Naberhuis et al.^18^ were stratified according to birth weight such that preterm pigs with the highest birth weight were compared to term pigs with the lowest birth weight. Following stratification by birth weight, the birth weight of the preterm pig subset was not different from the term pig subset (1035 versus 993 ± 54 g, *P* > 0.10). Relative body weight gain in the stratified group of animals was lower in preterm pigs than in term pigs (23.1 versus 35.4 ± 1.7 g·kg^–1^·d^–1^, *P* < 0.001) despite similar birth weight and equivalent nutrient intakes (i.e., relative to body weight).

**Fig. 2:**
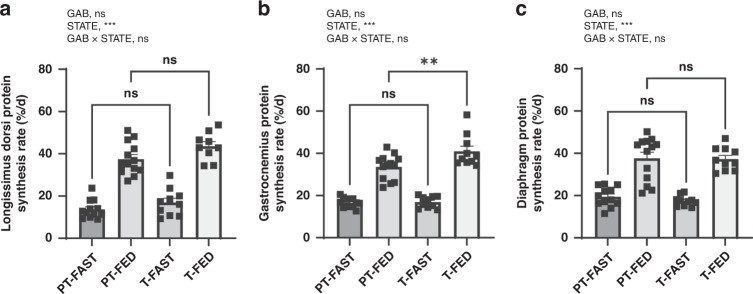
Fractional protein synthesis rate of skeletal muscles in a subset of preterm (PT) and term pigs (T) that did not differ in birth weight. Longissimus dorsi (**a**), gastrocnemius (**b**), and diaphragm (**c**) muscles from pigs were sampled in the fasted state (FAST) or 60 min after feeding (FED). Data were analyzed by two-factor ANOVA followed by the Tukey procedure; not all means comparisons are shown. Values are least-squares means ± SE; individual data are shown; PT-FAST, *n* = 12 (8 male and 4 female); PT-FED, *n* = 13 (9 male and 4 female); T-FAST, *n* = 10 (4 male and 6 female); T-FED, *n* = 10 (6 male and 4 female). GAB gestational age at birth (preterm or term), STATE feeding status (fasted or fed), ns not significant; *P < 0.05; **P < 0.01; ***P < 0.001.

Fractional protein synthesis rates in the LD, gastrocnemius, and diaphragm muscles were not correlated with birth weight in any treatment (Table 1). When data were stratified by birth weight, feeding increased protein synthesis in the LD, gastrocnemius, and diaphragm muscles (*P* < 0.001; Fig. 2). Protein synthesis in the gastrocnemius muscle was 18% lower in preterm compared to term pigs after feeding (*P* < 0.05) but was not significantly lower in the LD muscle of the preterm than term after a meal (*P* = 0.15). Protein synthesis in the diaphragm was not different among groups (*P* > 0.10).

**Table 1:** Pearson correlations between birth weight and skeletal muscle fractional protein synthesis rates in all preterm and term pigs among treatments.

GAB	STATE	Longissimus dorsi protein synthesis		Gastrocnemius protein synthesis		Diaphragm protein synthesis	
		*r*	*P* value	*r*	*P* value	*r*	*P* value
Preterm	Fasted	–0.22	ns	0.34	ns	0.26	ns
	Fed	0.23	ns	0.09	ns	–0.11	ns
Term	Fasted	0.08	ns	–0.23	ns	–0.37	ns
	Fed	–0.10	ns	–0.16	ns	–0.09	ns

PT-FAST, *n* = 23 (13 male and 10 female); PT-FED, *n* = 25 (14 male and 11 female); T-FAST, *n* = 21 (10 male and 11 female); T-FED, *n* = 21 (16 male and 5 female).

*GAB* gestational age at birth (i.e., preterm or term), *STATE* treatment condition (i.e., fasted or fed), *PT-FAST* preterm pigs in fasted state, *PT-FED* preterm pigs in fed state, *T-FAST* term pigs in fasted state, *T-FED* term pigs in fed state, *ns* not significant.

**Fig. 3:**
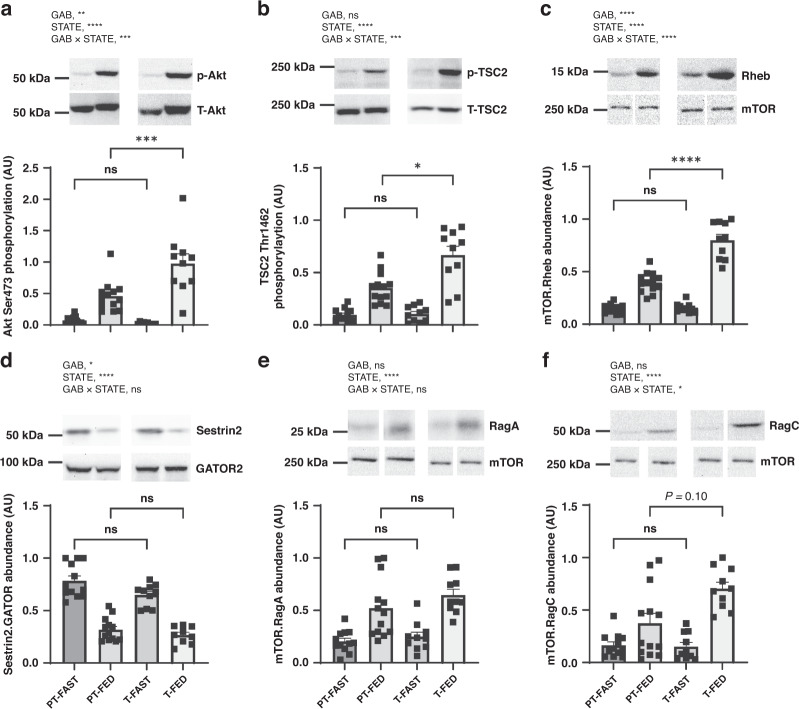
Insulin and amino acid signaling upstream of mTORC1 in longissimus dorsi muscle in a subset of preterm (PT) and term (T) pigs that did not differ in birth weight. Relative abundance of phosphorylated Akt Ser473 (**a**), phosphorylated TSC2 Thr1462 (**b**), mTOR·Rheb complex abundance (**c**), Sestrin2·GATOR2 complex abundance (**d**), mTOR·RagA complex abundance (**e**), and mTOR·RagC complex abundance (**f**) in longissimus dorsi muscle of a subset of preterm (PT) and term pigs (T) that did not differ in birth weight. Muscles from pigs were sampled in the fasted state (FAST) or 60 min after feeding (FED). White lines between bands indicate where images from the same blots were spliced to adjust sample order on the membrane for presentation. Data were analyzed by two-factor ANOVA followed by the Tukey procedure; not all means comparisons are shown. Values are least-squares means ± SE; individual data are shown; PT-FAST, *n* = 12 (8 male and 4 female); PT-FED, *n* = 13 (9 male and 4 female); T-FAST, *n* = 10 (4 male and 6 female); T-FED, *n* = 10 (6 male and 4 female). AU arbitrary units, GAB gestational age at birth (preterm or term), p phosphorylated, STATE feeding status (fasted or fed), T total, ns not significant; *P < 0.05; **P < 0.01; ***P < 0.001; ****P < 0.0001.

**Fig. 4:**
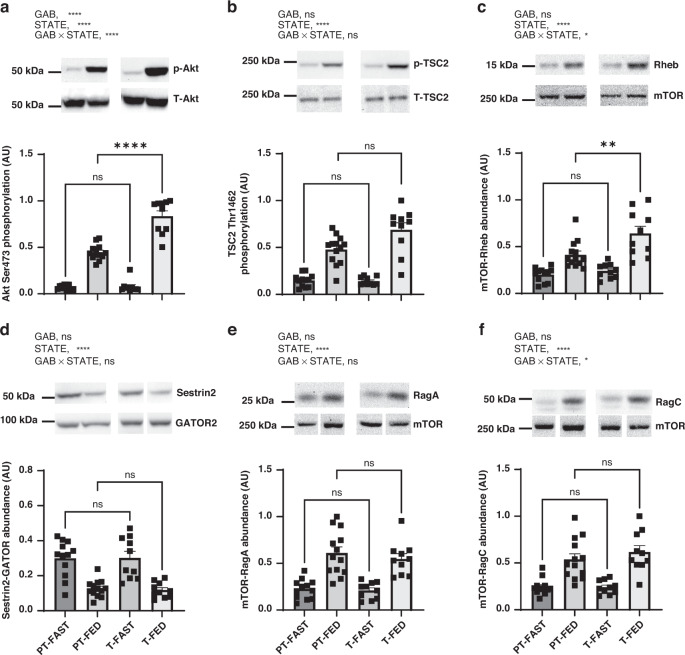
Insulin and amino acid signaling upstream of mTORC1 in gastrocnemius muscle in a subset of preterm (PT) and term (T) pigs that did not differ in birth weight. Relative abundance of phosphorylated Akt Ser473 (**a**), phosphorylated TSC2 Thr1462 (**b**), mTOR·Rheb complex abundance (**c**), Sestrin2·GATOR2 complex abundance (**d**), mTOR·RagA complex abundance (**e**), and mTOR·RagC complex abundance (**f**) in gastrocnemius muscle of a subset of preterm (PT) and term pigs (T) that did not differ in birth weight. Muscles from pigs were sampled in the fasted state (FAST) or 60 min after feeding (FED). Representative blots are shown; original and unedited blots are provided in Supplementary Fig. S2. White lines between bands indicate where images from the same blots were spliced to adjust sample order on the membrane for presentation. Data were analyzed by two-factor ANOVA followed by the Tukey procedure; not all means comparisons are shown. Values are least-squares means ± SE; individual data are shown; PT-FAST, *n* = 12 (8 male and 4 female); PT-FED, *n* = 13 (9 male and 4 female); T-FAST, *n* = 10 (4 male and 6 female); T-FED, *n* = 10 (6 male and 4 female). AU arbitrary units, GAB gestational age at birth (preterm or term), p phosphorylated, STATE feeding status (fasted or fed), T total, ns not significant; *P < 0.05; **P < 0.01; ****P < 0.0001.

The feeding-induced activation of signaling components that modulate insulin- and amino acid-dependent mTOR activity was altered by prematurity in LD (Fig. 3 and Table 2) and gastrocnemius (Fig. 4) muscles in the subset of preterm and term pigs stratified by birth weight. For insulin signaling in LD muscle, the feeding-induced increase in Akt phosphorylation on both Ser473 (Fig. 3) and Thr308 (Table 2), TSC2 phosphorylation, and mTOR·Rheb abundance (Fig. 3) was lower in preterm than in term pigs (*P* < 0.01). Insulin receptor phosphorylation, IRS1 phosphorylation, and IRS1·PI3K complex abundance were also enhanced with feeding (*P* < 0.001), but there was no difference between preterm and term pigs (Table 3). For amino acid signaling to mTORC1, the abundance of the inhibitory Sestrin2·GATOR2 complex^24^ was higher overall in preterm than in term pigs (*P* < 0.05); however, there was no interaction between GAB and feeding state, indicating that the abundance of the Sestrin2·GATOR2 complex was not different between preterm and term pigs after feeding (Fig. 3). Although feeding elicited an increase in mTOR·RagA and mTOR·RagC abundance in both preterm and term pigs, mTOR·RagA abundance was not different (*P* > 0.10) and mTOR·RagC (*P* = 0.10) abundance tended to be lower in preterm pigs after feeding. For insulin signaling in the gastrocnemius muscle, the feeding-induced increase in Akt phosphorylation and mTOR·Rheb abundance (*P* < 0.05) was lower in preterm than in term pigs (*P* < 0.01), whereas TSC2 phosphorylation tended to be lower in preterm than in term pigs after feeding (*P* = 0.10; Fig. 4). Conversely, amino acid signaling in the gastrocnemius muscle, while responsive to feeding, was neither different between preterm and term pigs in the fasted state nor in the fed state (*P* > 0.10).

**Table 2:** Relative phosphorylation or abundance (AU) of proteins and protein complexes involved in insulin signaling toward mTOR, translation initiation and elongation, and protein degradation in LD muscle of a subset of preterm and term pigs that did not differ in birth weight.

	Preterm		Term		*P* value		
	Fasted	Fed	Fasted	Fed	GAB	STATE	GAB × STATE
LD insulin signaling							
p-IR Tyr1185	0.09 ± 0.07	0.50 ± 0.07	0.07 ± 0.07	0.62 ± 0.07	ns	***	ns
p-IRS1 (Tyr)	0.26 ± 0.10	0.56 ± 0.10	0.17 ± 0.11	0.57 ± 0.11	ns	***	ns
IRS1·PI3K	0.34 ± 0.07	0.65 ± 0.07	0.28 ± 0.08	0.59 ± 0.08	ns	***	ns
p-Akt Thr308	0.02 ± 0.07^c^	0.37 ± 0.07^b^	0.04 ± 0.08^c^	0.79 ± 0.08^a^	ns	***	***
LD translation signaling							
p-eIF2α Ser51	0.63 ± 0.06	0.60 ± 0.06	0.61 ± 0.07	0.58 ± 0.07	ns	ns	ns
p-eEF2 Thr56	1.18 ± 0.40	1.45 ± 0.40	2.01 ± 0.44	1.56 ± 0.43	ns	ns	ns
LD protein degradation signaling							
p-FoxO3 Ser253	0.54 ± 0.07	0.53 ± 0.07	0.46 ± 0.08	0.60 ± 0.08	ns	ns	ns
LC3-II:LC3	0.59 ± 0.10	0.15 ± 0.10	0.68 ± 0.11	0.07 ± 0.10	ns	***	ns
MuRF1	0.49 ± 0.12	0.55 ± 0.13	0.72 ± 0.14	0.72 ± 0.14	ns	ns	ns
Atrogin-1	0.54 ± 0.10	0.66 ± 0.10	0.77 ± 0.11	0.67 ± 0.11	ns	ns	ns

Values are least-square means ± SE calculated from two-factor ANOVA, PT-FAST, *n* = 12 (8 male and 4 female); PT-FED, *n* = 13 (9 male and 4 female); T-FAST, *n* = 10 (4 male and 6 female); T-FED, *n* = 10 (6 male and 4 female).

*GAB* gestational age at birth (i.e., preterm or term), *STATE* treatment condition (i.e., fasted or fed).

Labeled means in a row without a common superscript letter differ, *P* < 0.05; ns, not significant; ***P < 0.001.

**Table 3:** Fractional protein synthesis rate (%/d) and relative abundance of phosphorylated AKT, S6K1, 4EBP1 (AU) in select organs of a subset of preterm and term pigs that did not differ in birth weight.

	Preterm		Term		*P* value		
	Fasted	Fed	Fasted	Fed	GAB	STATE	GAB × STATE
Heart							
Protein synthesis	16.4 ± 1.5^c^	26.0 ± 1.5^b^	13.0 ± 1.7^c^	35.9 ± 1.6^a^	ns	***	***
p-AKT Ser473	0.09 ± 0.03^c^	0.39 ± 0.03^b^	0.09 ± 0.03^c^	0.87 ± 0.03^a^	***	***	***
p-S6K1 Thr389	0.08 ± 0.03^c^	0.40 ± 0.03^b^	0.09 ± 0.03^c^	0.78 ± 0.03^a^	***	***	***
p-4EBP1 Thr70	0.16 ± 0.03^c^	0.50 ± 0.03^b^	0.12 ± 0.03^c^	0.86 ± 0.03^a^	**	***	***
Lung							
Protein synthesis	22.6 ± 2.0	30.0 ± 2.0	19.2 ± 2.2	31.2 ± 2.1	ns	***	ns
p-AKT Ser473	0.16 ± 0.04	0.71 ± 0.04	0.14 ± 0.05	0.59 ± 0.04	ns	***	ns
p-S6K1 Thr389	0.09 ± 0.05	0.68 ± 0.05	0.08 ± 0.06	0.67 ± 0.06	ns	***	ns
p-4EBP1 Thr70	0.08 ± 0.04	0.52 ± 0.04	0.07 ± 0.05	0.48 ± 0.05	ns	***	ns
Liver							
Protein synthesis	62.5 ± 2.0	73.2 ± 1.8	51.5 ± 2.0	64.3 ± 2.0	***	***	ns
p-AKT Ser473	0.13 ± 0.04^c^	0.78 ± 0.04^a^	0.10 ± 0.05^c^	0.50 ± 0.05^b^	***	***	**
p-S6K1 Thr389	0.10 ± 0.04^c^	0.80 ± 0.04^a^	0.07 ± 0.04^c^	0.53 ± 0.04^b^	*	***	***
p-4EBP1 Thr70	0.10 ± 0.04^c^	0.72 ± 0.04^a^	0.09 ± 0.04^c^	0.52 ± 0.04^b^	*	***	*
Pancreas							
Protein synthesis	48.3 ± 4.7^c^	72.9 ± 4.8^b^	51.4 ± 5.3^c^	91.4 ± 5.2^a^	ns	***	**
p-AKT Ser473	0.13 ± 0.07^c^	0.43 ± 0.07^b^	0.13 ± 0.08^c^	0.73 ± 0.08^a^	ns	***	**
p-S6K1 Thr389	0.08 ± 0.04^c^	0.33 ± 0.04^b^	0.08 ± 0.04^c^	0.67 ± 0.04^a^	***	***	***
p-4EBP1 Thr70	0.14 ± 0.04^c^	0.53 ± 0.04^b^	0.15 ± 0.04^c^	0.80 ± 0.04^a^	***	***	**
Jejunum							
Protein synthesis	33.8 ± 3.9	47.0 ± 4.1	36.2 ± 4.4	53.1 ± 4.4	ns	***	ns
p-AKT Ser473	0.17 ± 0.05	0.57 ± 0.05	0.11 ± 0.05	0.69 ± 0.05	ns	***	ns
p-S6K1 Thr389	0.18 ± 0.06	0.56 ± 0.06	0.14 ± 0.07	0.56 ± 0.07	ns	***	ns
p-4EBP1 Thr70	0.15 ± 0.05	0.71 ± 0.05	0.13 ± 0.05	0.75 ± 0.05	ns	***	ns
Kidney							
Protein synthesis	29.7 ± 1.8	41.4 ± 1.8	32.2 ± 2.0	44.5 ± 2.0	ns	***	ns
p-AKT Ser473	0.10 ± 0.08	0.40 ± 0.08	0.13 ± 0.09	0.52 ± 0.08	ns	***	ns
p-S6K1 Thr389	0.08 ± 0.04^c^	0.46 ± 0.04^b^	0.12 ± 0.05^c^	0.80 ± 0.05^a^	*	***	***
p-4EBP1 Thr70	0.12 ± 0.04^c^	0.45 ± 0.04^b^	0.12 ± 0.05^c^	0.72 ± 0.05^a^	ns	***	***
Brain							
Protein synthesis	17.8 ± 1.7	28.1 ± 1.7	18.9 ± 1.9	26.5 ± 1.8	ns	***	ns
p-AKT Ser473	0.27 ± 0.09	0.73 ± 0.09	0.22 ± 010	0.70 ± 0.10	ns	***	ns
p-S6K1 Thr389	0.15 ± 0.05	0.62 ± 0.05	0.17 ± 0.05	0.64 ± 0.05	ns	***	ns
p-4EBP1 Thr70	0.14 ± 0.06	0.47 ± 0.06	0.13 ± 0.06	0.45 ± 0.06	ns	***	ns

Values are least-square means ± SE calculated from two-factor ANOVA, PT-FAST, *n* = 12 (8 male and 4 female); PT-FED, *n* = 13 (9 male and 4 female); T-FAST, *n* = 10 (4 male and 6 female); T-FED, *n* = 10 (6 male and 4 female).

*GAB* gestational age at birth (i.e., preterm or term), *STATE* treatment condition (i.e., fasted or fed), *PT-FAST* preterm pigs in fasted state, *PT-FED* preterm pigs in fed state, *T-FAST* term pigs in fasted state, *T-FED* term pigs in fed state.

Labeled means in a row without a common superscript letter differ, *P* < 0.05; ns, not significant; *P < 0.05; **P < 0.01; ***P < 0.001.

**Fig. 5:**
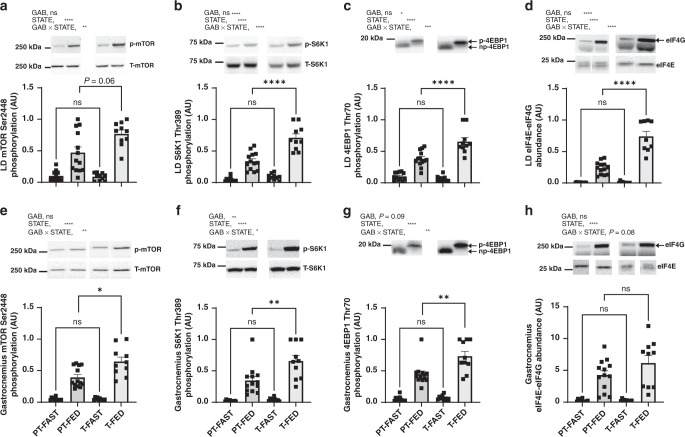
Downstream mTORC1 signaling in longissimus dorsi and gastrocnemius muscles in a subset of preterm (PT) and term (T) pigs that did not differ in birth weight. Relative abundance of phosphorylated mTOR Ser2448 (**a**), phosphorylated S6K1 Thr389 (**b**), phosphorylated 4EBP1 Thr70 (**c**), and eIF4E·eIF4G complex abundance (**d**) in longissimus dorsi muscle and phosphorylated mTOR Ser2448 (**e**), phosphorylated S6K1 Thr389 (**f**), phosphorylated 4EBP1 Thr70 (**g**), and eIF4E·eIF4G complex abundance (**h**) in gastrocnemius muscle of a subset of preterm (PT) and term pigs (T) that did not differ in birth weight. Muscles from pigs were sampled in the fasted state (FAST) or 60 min after feeding (FED). Representative blots are shown; original and unedited blots are provided in Supplementary Fig. S3. White lines between bands indicate where images from the same blots were spliced to adjust sample order on the membrane for presentation. Data were analyzed by two-factor ANOVA followed by the Tukey procedure; not all means comparisons are shown. Values are least-squares means ± SE; individual data are shown; PT-FAST, *n* = 12 (8 male and 4 female); PT-FED, *n* = 13 (9 male and 4 female); T-FAST, *n* = 10 (4 male and 6 female); T-FED, *n* = 10 (6 male and 4 female). AU arbitrary units, GAB gestational age at birth (preterm or term), np non-phosphorylated, p phosphorylated, STATE feeding status (fasted or fed), T total, ns not significant; *P < 0.05; **P < 0.01; ***P < 0.001; ****P < 0.0001.

**Fig. 6:**
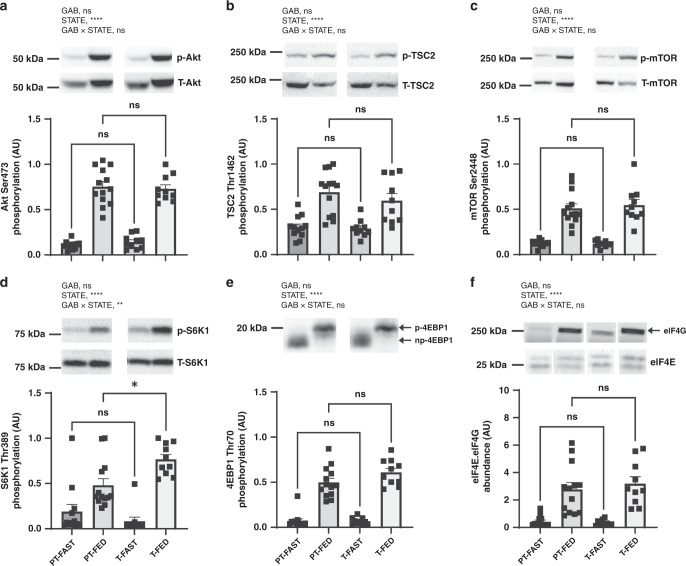
Insulin and amino acid signaling upstream of mTORC1 and downstream mTORC1 signaling in diaphragm muscle in a subset of preterm (PT) and term (T) pigs that did not differ in birth weight. Relative abundance of phosphorylated Akt Ser473 (**a**), phosphorylated TSC2 Thr1462 (**b**), phosphorylated mTOR Ser2448 (**c**), phosphorylated S6K1 Thr389 (**d**), phosphorylated 4EBP1 Thr70 (**e**), and eIF4E·eIF4G complex abundance (**f**) in diaphragm muscle of a subset of preterm (PT) and term pigs (T) that did not differ in birth weight. Muscles from pigs were sampled in the fasted state (FAST) or 60 min after feeding (FED). Representative blots are shown; original and unedited blots are provided in Supplementary Fig. S4. White lines between bands indicate where images from the same blots were spliced to adjust sample order on the membrane for presentation. Data were analyzed by two-factor ANOVA followed by the Tukey procedure; not all means comparisons are shown. Values are least-squares means ± SE; individual data are shown; PT-FAST, *n* = 12 (8 male and 4 female); PT-FED, *n* = 13 (9 male and 4 female); T-FAST, *n* = 10 (4 male and 6 female); T-FED, *n* = 10 (6 male and 4 female). AU arbitrary units, GAB gestational age at birth (preterm or term), np non-phosphorylated, p phosphorylated, STATE feeding status (fasted or fed), T total, ns not significant; *p < 0.05; **P < 0.01; ****P < 0.0001.

Prematurity attenuated translation initiation signaling in LD and gastrocnemius muscles in the stratified group of animals (Fig. 5). The feeding-induced increase in mTOR phosphorylation of Ser2448 tended to be lower in LD muscle (*P* = 0.06), whereas mTOR phosphorylation was lower in gastrocnemius muscle (*P* < 0.05), in preterm compared to term pigs. Although 4EBP1 and S6K1 phosphorylation and eIF4E·eIF4G complex abundance in LD muscle increased after feeding in both preterm and term pigs (*P* < 0.001), 4EBP1, S6K1, and eIF4E·eIF4G activation were all lower in preterm compared to term pigs after feeding (*P* < 0.001). The activation state of 4EBP1 and S6K1, but not eIF4E·eIF4G, in gastrocnemius muscle mirrored that in the LD muscle (*P* < 0.01). Although the feeding-induced phosphorylation of Akt, TSC2, mTOR, and 4EBP1 and assembly of eIF4E·eIF4G in the diaphragm muscle was not altered by prematurity (*P* > 0.10; Fig. 6), the feeding-induced increase in S6K1 phosphorylation was lower in preterm than in term pigs (*P* < 0.05).

The phosphorylation of eIF2α and eEF2 in LD muscle did not differ among groups (Table 2). Indirect markers of myofibrillar protein degradation, including FoxO3 phosphorylation, MuRF1 abundance, and Atrogin-1 abundance did not differ among treatments in LD muscle.^25,26^ The ratio of LC3-II to total LC3, an index of autophagosome formation,^27^ declined with feeding in LD muscle (*P* < 0.001) but there was no difference between preterm and term pigs.

In the subset of preterm and term pigs stratified by birth weight, liver protein synthesis was higher in preterm than in term pigs (*P* < 0.001; Table 3). While protein synthesis rates increased with feeding in all organs (*P* < 0.001), protein synthesis increased 38% more in the heart (*P* < 0.001) and 25% more in the pancreas (*P* = 0.05) after feeding in term compared to preterm pigs. The pattern in Akt, S6K1, and 4EBP1 phosphorylation largely corresponded to the feeding-induced increase in fractional protein synthesis across organs.

## Discussion

Premature birth accounts for approximately 1 in 10 live births in the United States.^28^ While survival rates for premature infants now exceed 90% due to advances in perinatal care, many of these infants are at risk for postnatal growth faltering.^29–31^ Growth faltering in premature infants is largely attributed to diminished lean tissue accretion, especially in skeletal muscle, whereas fat accretion is maintained or even enhanced.^2,12,32^ Previously, we reported that, among skeletal muscles in preterm pigs, LD and gastrocnemius, but not diaphragm, were resistant to the feeding-induced stimulation of mTORC1 activation and protein synthesis.^18^ Among organs, protein synthesis in the heart and pancreas were likewise less responsive to feeding in preterm pigs. However, we did not discern whether the lower birth weight of preterm pigs contributes to the observed anabolic resistance in these tissues, especially in skeletal and cardiac muscle. The absence of any correlation between birth weight and relative body weight gain in the current study implies that factors other than birth weight contribute to the observed differences in translation initiation and protein synthesis between preterm and term pigs.

Protein synthesis in the neonate is highly responsive to insulin- and amino acid-induced activation of mTORC1.^22^ The disconnect between insulin signaling in LD muscle upstream of Akt and downstream of Akt in the current study was consistent with our previous analysis of all pigs.^18^ Upstream of Akt, IR phosphorylation, IRS1 phosphorylation, and IRS1·PI3K abundance was not affected by prematurity in our previous analysis and current secondary analysis of preterm and term pigs with similar birth weight. Insulin signaling downstream of Akt, including Akt phosphorylation on both Thr308 and Ser473 needed for maximal Akt activity,^33^ TSC2 phosphorylation, and mTOR·Rheb abundance, was blunted by prematurity in LD muscle in both the primary and secondary analyses. Unlike the previous analysis, however, Sestrin2·GATOR2 abundance was higher overall in LD muscle of preterm compared to term pigs of similar birth weight. Leucine binds Sestrin2 and disrupts the interaction between Sestrin2 and GATOR2, enabling GATOR2 to block GATOR1-mediated inhibition of mTOR·RagA and mTORC1 assembly.^34^ Our results suggest that the clear resistance of LD muscle to insulin and marginal baseline resistance to leucine, and amino acids more broadly, does not fully translate to a reduction in LD muscle protein synthesis.

Insulin signaling in the gastrocnemius muscle, from Akt phosphorylation through Rheb-mediated mTOR activation,^35^ was blunted in preterm pigs after feeding. Despite the lack of apparent effect of prematurity on the response of the gastrocnemius muscle to elevated plasma amino acid levels, however, translation initiation signaling and protein synthesis in the gastrocnemius muscle were lower in preterm than in term pigs. The altered activation state of most signaling proteins in both preterm LD and gastrocnemius muscles points toward some resistance to feeding-induced anabolism. However, the activation of the amino acid signaling pathway does not appear to be disrupted to the same extent as the insulin signaling pathway in the skeletal muscle of the preterm neonate. Insulin-mediated mTORC1 activation in the diaphragm, on the other hand, is not affected by prematurity. The molecular basis for the inconsistent response among skeletal muscles in the current study is unclear but could reflect preferential protein accretion in muscles critical for postnatal survival. It is also possible that a disproportionate mechanical load on the diaphragm, relative to other skeletal muscles, overcomes any intrinsic deficit elicited by preterm birth, muscle disuse, or both on mTORC1 activation.^36^ Collectively, the absence of any correlation between birth weight and fractional protein synthesis rates in any skeletal muscle supports the hypothesis that the difference in protein synthesis between preterm and term pigs after feeding is a consequence of gestational age and muscle immaturity at birth rather than birth weight and that the observed attenuation is linked to impaired insulin signaling.

Postnatal lean growth is ascribed to protein accretion in both skeletal muscle and solid organs. Among organs, only protein synthesis in the heart, liver, and pancreas was affected by GAB or an interaction between GAB and feeding status in preterm pigs and term pigs with similar birth weights. Apart from minor differences in the phosphorylation of several signaling proteins downstream of mTORC1 among treatments, these results were consistent with our previous analysis that included these data from all pigs and also mirrored trends in tissue protein synthesis rates.^18^ The premature heart, while resistant to the feeding-induced activation of mTORC1 and protein synthesis, is one of the first organs to develop during gestation.^37^ However, the heart is especially susceptible to prematurity-induced alterations to its geometry and function, such as a thinner left ventricle wall,^38^ consistent with our observation that preterm birth also reduces protein synthesis in the heart after feeding independently of birth weight. Conversely, preterm and term cardiac muscle protein synthesis increases equally in response to insulin stimulation during a hyperinsulinemic-euaminoacidemic-euglycemic clamp and to amino acid stimulation during a euinsulinemic-hyperaminoacidemic-euglycemic clamp.^19^ The basis for this discrepancy is unclear but suggests that the effect of insulin and amino acids is additive in the preterm heart, whereas the effect is synergistic in the term heart. The higher rate of hepatic protein synthesis in preterm compared to term pigs may reflect the high but declining synthetic capacity of the liver for albumin synthesis across gestation,^39^ and the lower rate of pancreatic protein synthesis in preterm compared to term pigs after feeding is consistent with reports that preterm sheep have reduced pancreatic β-cell mass and less glucose-stimulated insulin secretion at 4 weeks term-equivalent age.^40^ Whether these differences in organ protein synthesis rates between preterm and term pigs are linked to long-term functional consequences is unclear. Indeed, prematurity is associated with an increased risk of developing metabolic syndrome and cardiovascular disease, in which these organs have significant roles.^4^ Lower stroke volume and reduced cardiac output during exercise in adolescents born preterm suggest that deficits in the capacity of the heart, and perhaps other organs, to respond to anabolic signals may persist.^41^

The current secondary analysis of data from a subset of preterm and term pigs with similar birth weights indicates that the blunted response to feeding of the insulin and amino acid signaling pathways that lead to translation initiation and protein synthesis in the skeletal muscle of preterm pigs is independent of body weight and likely contributes to reduced body weight gain. These results suggest that the mTORC1 pathway is regulated in a coordinated manner at the whole-body level during early development to partition nutrients to tissues critical for postnatal survival (i.e., from axial and limb muscles to the diaphragm and most solid organs). The heart, however, appears to be an exception to this general paradigm and points toward a divergent response to anabolic stimuli in skeletal and cardiac muscles. While the selection of high birth weight preterm pigs and low birth weight term pigs may bias our observations, we would anticipate in this context that the response to feeding would favor preterm compared to term pigs because preterm pigs with low birth weight and term pigs with high birth weight are excluded from the analysis. Yet, the growth rate in preterm pigs was still lower than in term pigs despite comparable birth weight and equivalent nutrient intake. Indeed, cumulative deficits in energy and protein intake have been estimated to account for up to 45% of the reduction in body weight z-scores in very preterm infants,^42^ although this was unlikely to be the case in the current study. Overall, this implies that prematurity, and not low birth weight, reduces the capacity of skeletal muscle, apart from the diaphragm, and cardiac muscle to promote mTORC1 activation and protein synthesis after feeding and contributes to lower rates of lean mass accretion in premature infants.^2^ A persistent lower capacity to use nutrients for muscle growth may favor the development of adiposity in infants born preterm and during accelerated catch-up growth later in infancy.^43^

## Supplementary information

Supplementary Information

## Data Availability

The datasets generated during and/or analyzed during the current study are available from the corresponding author on reasonable request.
