# Supplementary material for: Prematurity blunts protein synthesis in skeletal muscle independently of body weight in neonatal pigs
Source: Pediatr Res. 2023 Jan 10;94(1):143–52. doi: 10.1038/s41390-022-02456-3 (PMC10330946; doi:10.1038/s41390-022-02456-3)
Supplement: Supplementary file 1 — Supplementary Information [file 41390_2022_2456_MOESM1_ESM.pdf]

Supplemental Figure S1. Original unedited chemiluminescent blots of p-Akt Ser473 and T-Akt (A), p-TSC2 Thr1462 and T-TSC2 (B), Rheb and mTOR (C), Sestrin2 and GATOR2 (D), RagA and mTOR (E), and RagC and mTOR (F) in longissimus dorsi muscle. Red outline indicates representative blot images included in manuscript Figure 3. Sample number 1-12, PT-FAST; sample number 13-25, PT-FED; sample number 26-35, T-FAST; sample number 36-45, T-FED. Abbreviations used: p, phosphorylated; T, total; PT, preterm; T, term.

Supplemental Figure S2. Original unedited chemiluminescent blots of p-Akt Ser473 and T-Akt (A), p-TSC2 Thr1462 and T-TSC2 (B), Rheb and mTOR (C), Sestrin2 and GATOR2 (D), RagA and mTOR (E), and RagC and mTOR (F) in gastrocnemius muscle. Red outline indicates representative blot images included in manuscript Figure 4. Sample number 1-12, PT-FAST; sample number 13-25, PT-FED; sample number 26-35, T-FAST; sample number 36-45, T-FED. Abbreviations used: p, phosphorylated; T, total; PT, preterm; T, term.

Supplemental Figure S3. Original unedited chemiluminescent blots of p-mTOR Ser2448 and total mTOR (A), p-S6K1 Thr389 and T-S6K1 (B), 4EBP1 (C), and eIF4E·eIF4G (D) in longissimus dorsi muscle and p-mTOR Ser2448 and total mTOR (E), p-S6K1 Thr389 and T-S6K1 (F), 4EBP1 (G), and eIF4E·eIF4G (H) in gastrocnemius muscle. Red outline indicates representative blot images included in manuscript Figure 5. Sample number 1-12, PT-FAST; sample number 13-25, PT-FED; sample number 26-35, T-FAST; sample number 36-45, T-FED. Abbreviations used: p, phosphorylated; T, total; PT, preterm; T, term.

Supplemental Figure S4. Original unedited chemiluminescent blots of p-Akt Ser473 and T-Akt (A), p-TSC2 Thr1462 and T-TSC2 (B), p-mTOR Ser2448 and total mTOR (C), p-S6K1 Thr389 and T-S6K1 (D), 4EBP1 (E), and eIF4E·eIF4G (F) in diaphragm muscle. Red outline indicates representative blot images included in manuscript Figure 6. Sample number 1-12, PT-FAST; sample number 13-25, PT-FED; sample number 26-35, T-FAST; sample number 36-45, T-FED. Abbreviations used: p, phosphorylated; T, total; PT, preterm; T, term.

Fig. S1 A

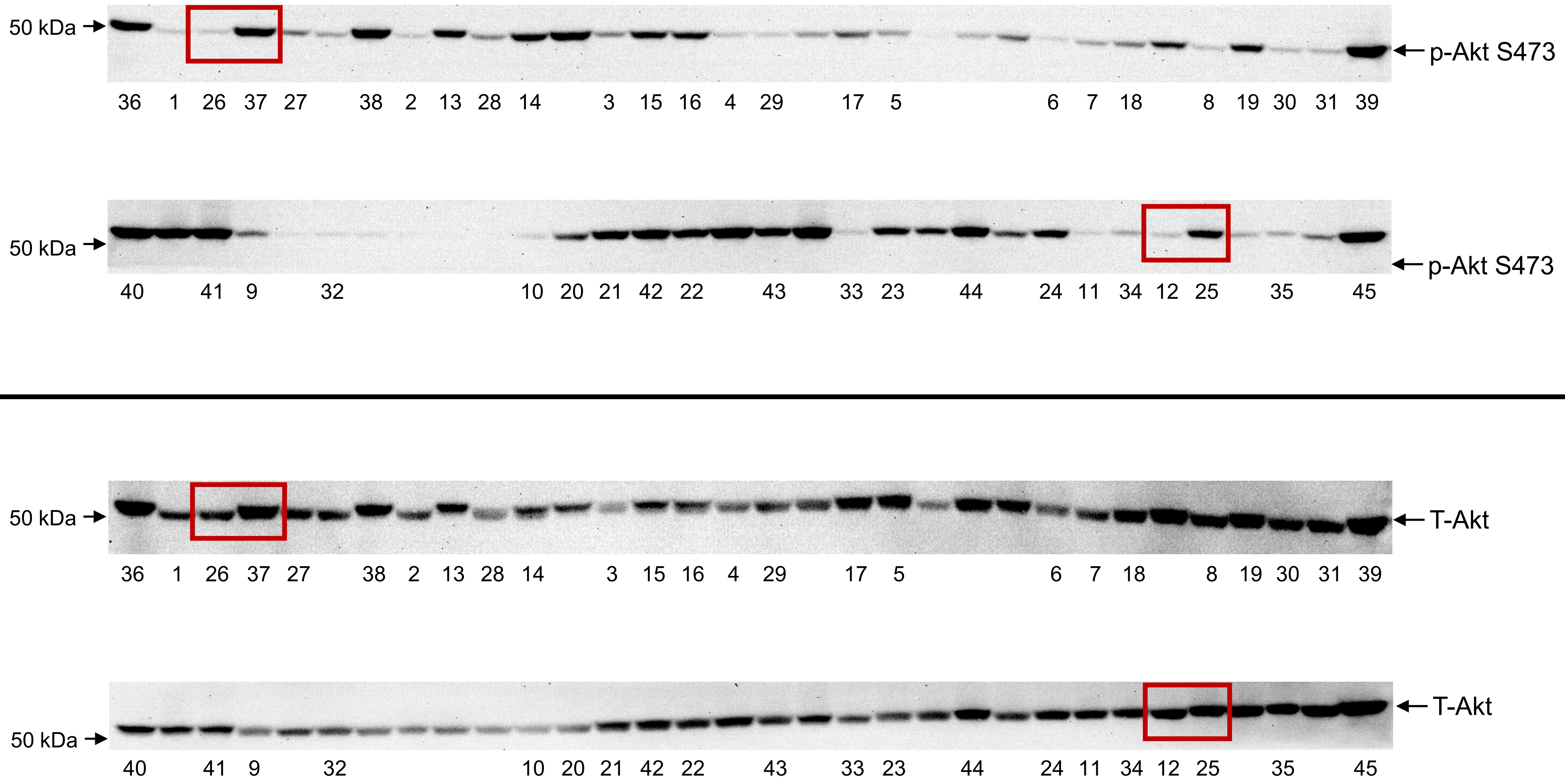

50 kDa
p-Akt S473
36 1 26 37 27 38 2 13 28 14 3 15 16 4 29 17 5 6 7 18 8 19 30 31 39
50 kDa
p-Akt S473
40 41 9 32 10 20 21 42 22 43 33 23 44 24 11 34 12 25 35 45
50 kDa
T-Akt
36 1 26 37 27 38 2 13 28 14 3 15 16 4 29 17 5 6 7 18 8 19 30 31 39
50 kDa
T-Akt
40 41 9 32 10 20 21 42 22 43 33 23 44 24 11 34 12 25 35 45

Fig. S1 B

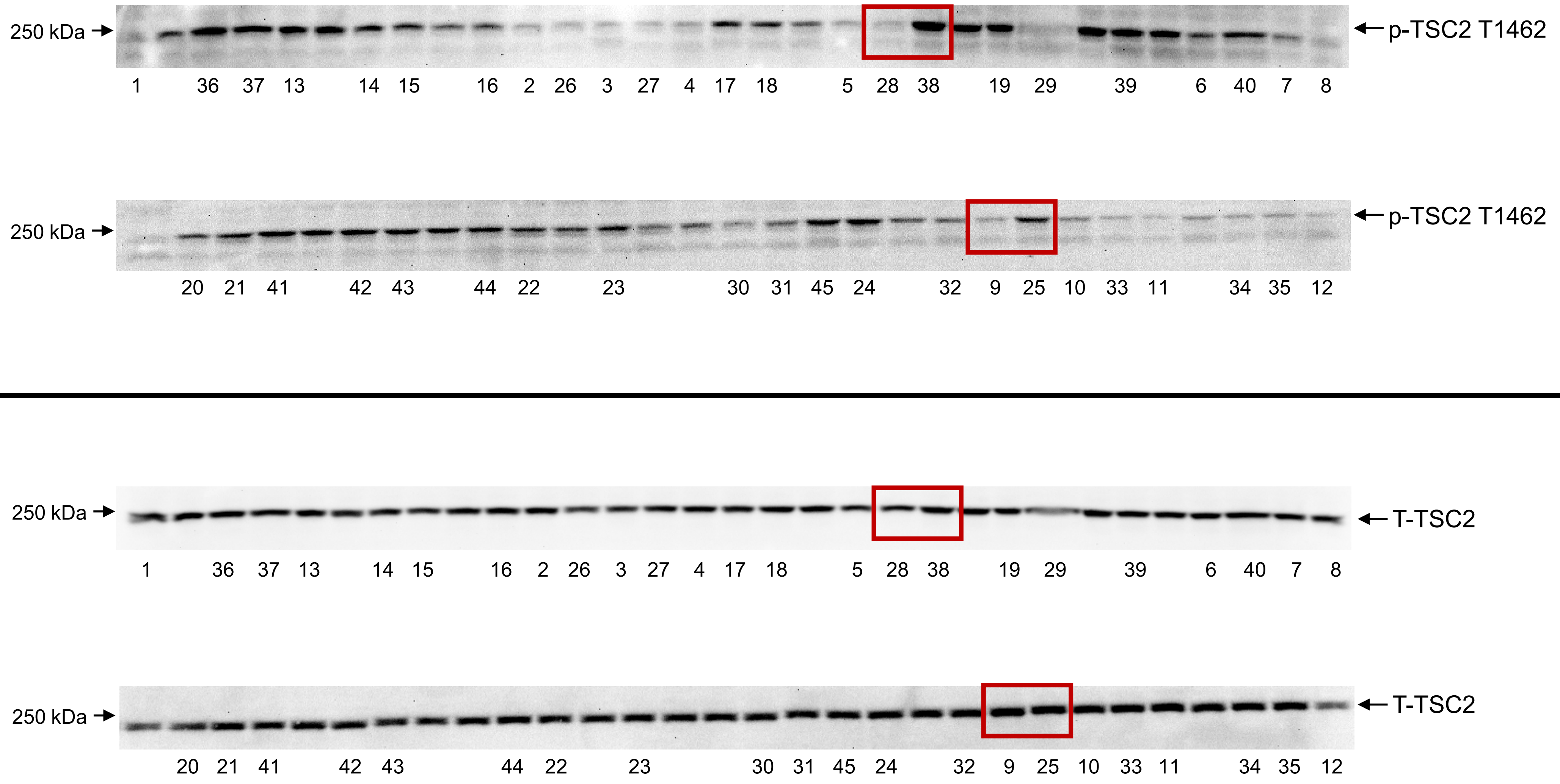
250 kDa
p-TSC2 T1462
1 36 37 13 14 15 16 2 26 3 27 4 17 18 5 28 38 19 29 39 6 40 7 8
250 kDa
p-TSC2 T1462
20 21 41 42 43 44 22 23 30 31 45 24 32 9 25 10 33 11 34 35 12
250 kDa
T-TSC2
1 36 37 13 14 15 16 2 26 3 27 4 17 18 5 28 38 19 29 39 6 40 7 8
250 kDa
T-TSC2
20 21 41 42 43 44 22 23 30 31 45 24 32 9 25 10 33 11 34 35 12

Fig. S1 C

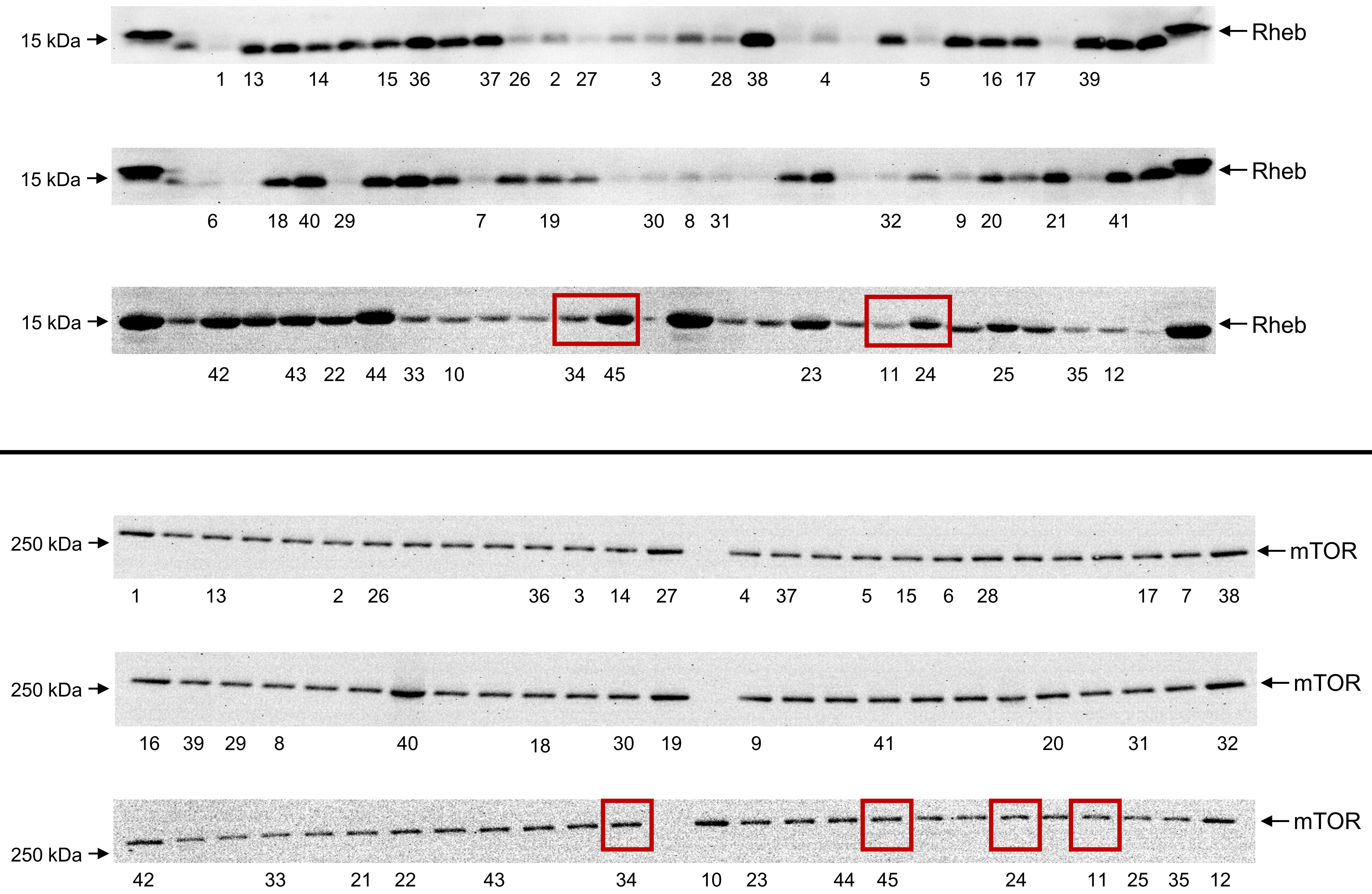
15 kDa
Rheb
1 13 14 15 36 37 26 2 27 3 28 38 4 5 16 17 39
15 kDa
Rheb
6 18 40 29 7 19 30 8 31 32 9 20 21 41
15 kDa
Rheb
42 43 22 44 33 10 34 45 23 11 24 25 35 12
250 kDa
mTOR
1 13 2 26 36 3 14 27 4 37 5 15 6 28 17 7 38
250 kDa
mTOR
16 39 29 8 40 18 30 19 9 41 20 31 32
250 kDa
mTOR
42 33 21 22 43 34 10 23 44 45 24 11 25 35 12

Fig. S1 D

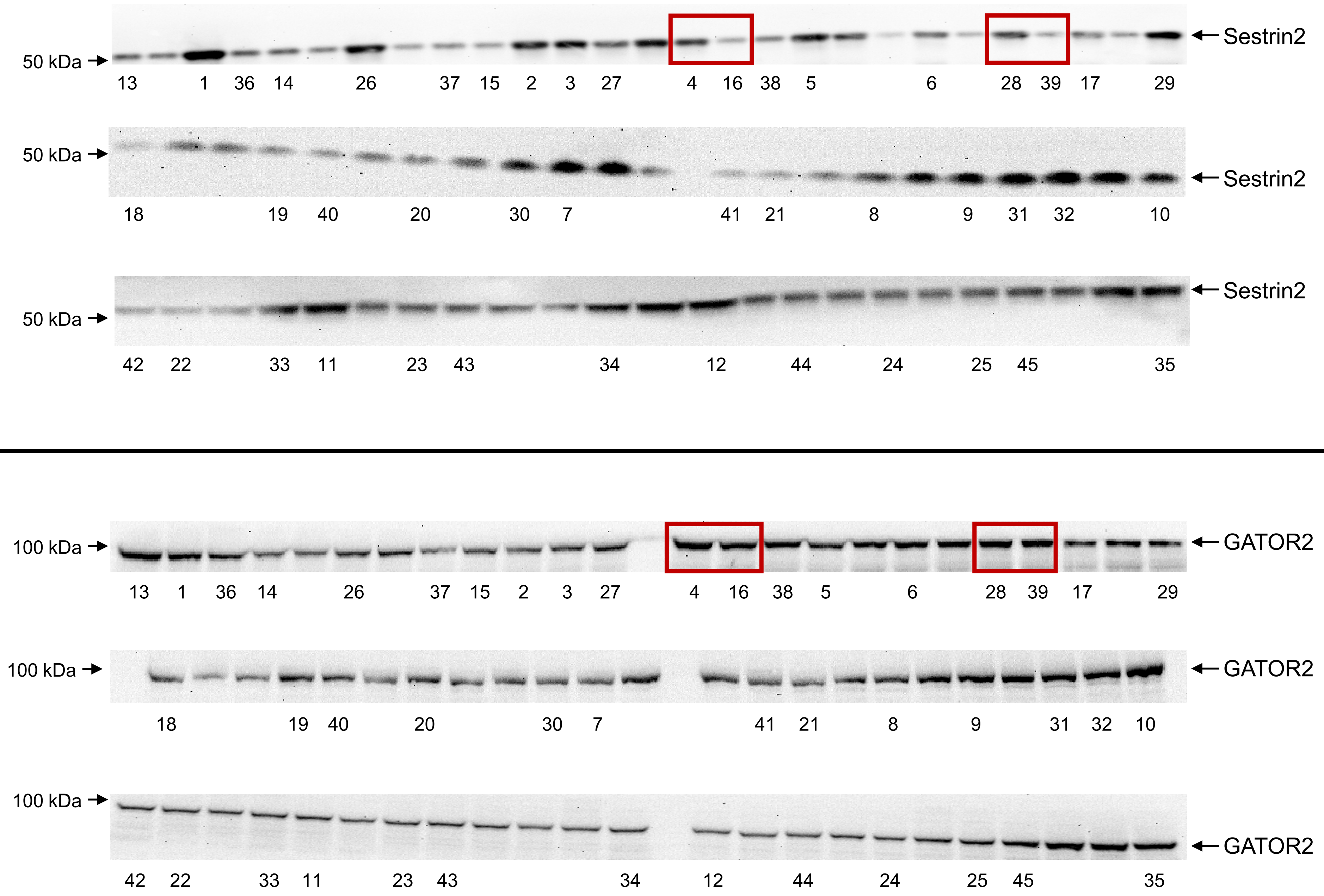
50 kDa
Sestrin2
13 1 36 14 26 37 15 2 3 27 4 16 38 5 6 28 39 17 29
50 kDa
Sestrin2
18 19 40 20 30 7 41 21 8 9 31 32 10
Sestrin2
50 kDa
42 22 33 11 23 43 34 12 44 24 25 45 35
100 kDa
GATOR2
13 1 36 14 26 37 15 2 3 27 4 16 38 5 6 28 39 17 29
100 kDa
GATOR2
18 19 40 20 30 7 41 21 8 9 31 32 10
100 kDa
GATOR2
42 22 33 11 23 43 34 12 44 24 25 45 35

Fig. S1 E

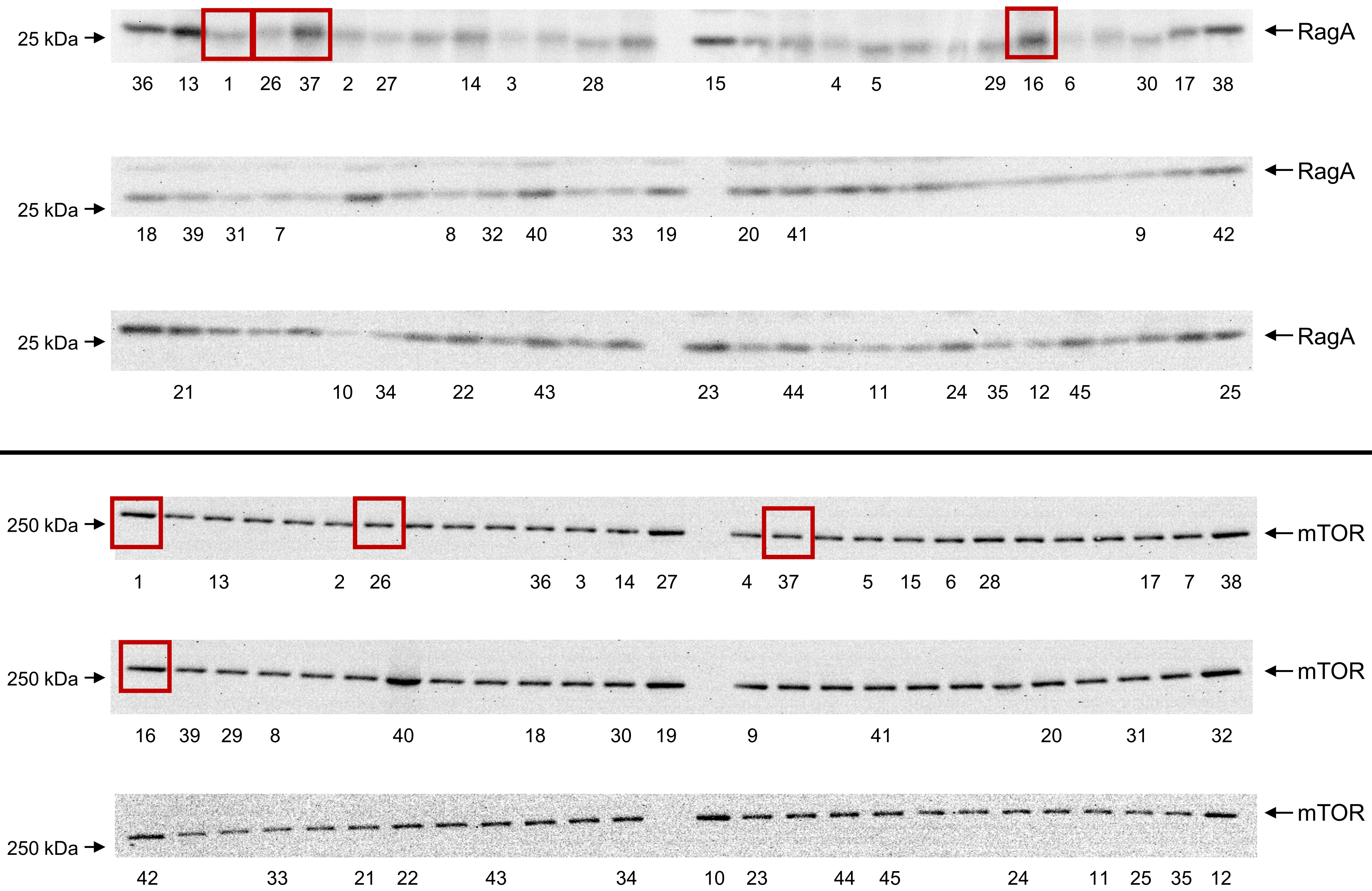
25 kDa
RagA
36 13 1 26 37 2 27 14 3 28 15 4 5 29 16 6 30 17 38
25 kDa
RagA
18 39 31 7 8 32 40 33 19 20 41 9 42
25 kDa
RagA
21 10 34 22 43 23 44 11 24 35 12 45 25
250 kDa
mTOR
1 13 2 26 36 3 14 27 4 37 5 15 6 28 17 7 38
250 kDa
mTOR
16 39 29 8 40 18 30 19 9 41 20 31 32
250 kDa
mTOR
42 33 21 22 43 34 10 23 44 45 24 11 25 35 12

Fig. S1 F

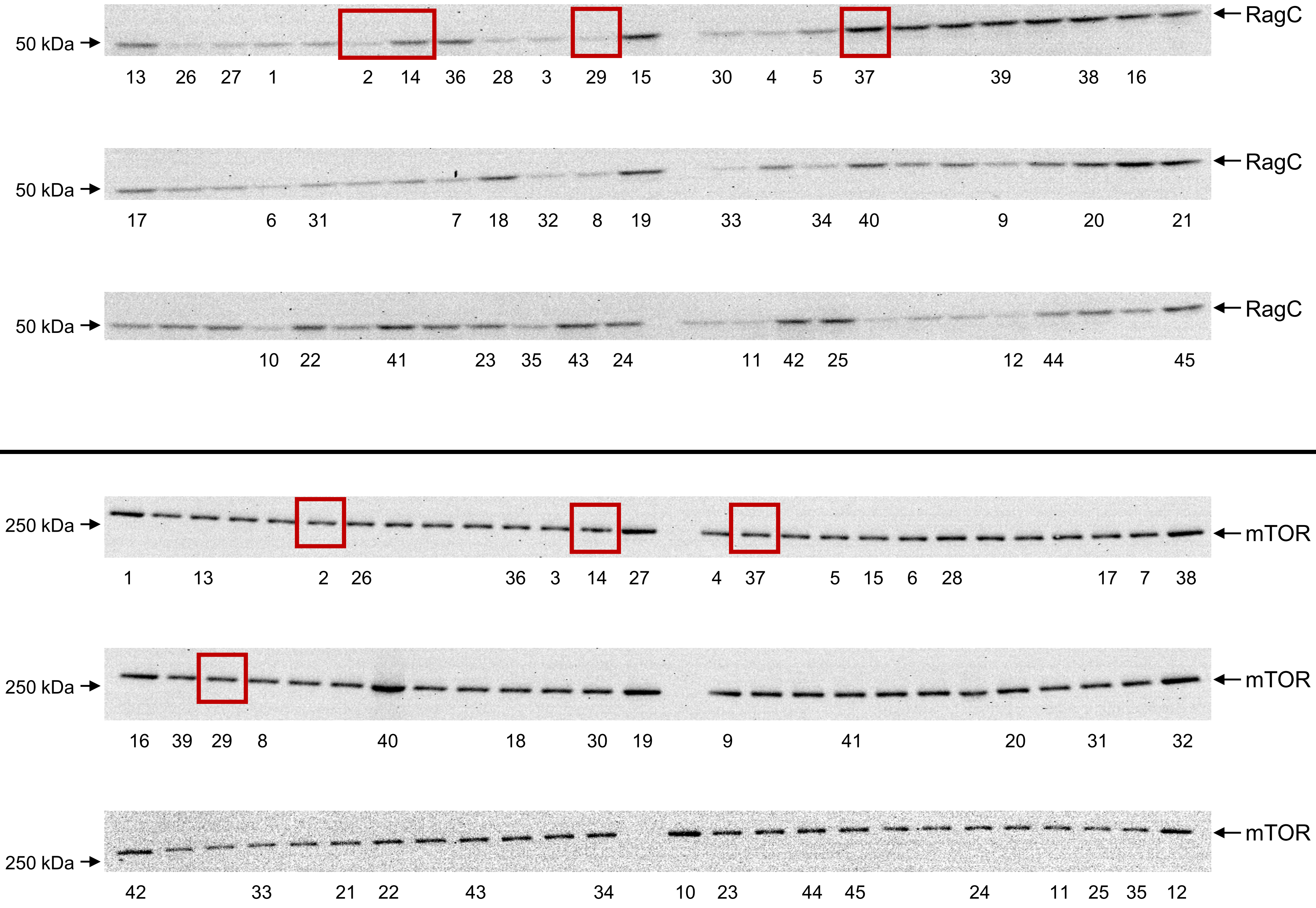
50 kDa
RagC
13 26 27 1 2 14 36 28 3 29 15 30 4 5 37 39 38 16
50 kDa
RagC
17 6 31 7 18 32 8 19 33 34 40 9 20 21
50 kDa
RagC
10 22 41 23 35 43 24 11 42 25 12 44 45
250 kDa
mTOR
1 13 2 26 36 3 14 27 4 37 5 15 6 28 17 7 38
250 kDa
mTOR
16 39 29 8 40 18 30 19 9 41 20 31 32
250 kDa
mTOR
42 33 21 22 43 34 10 23 44 45 24 11 25 35 12

Fig. S2 A

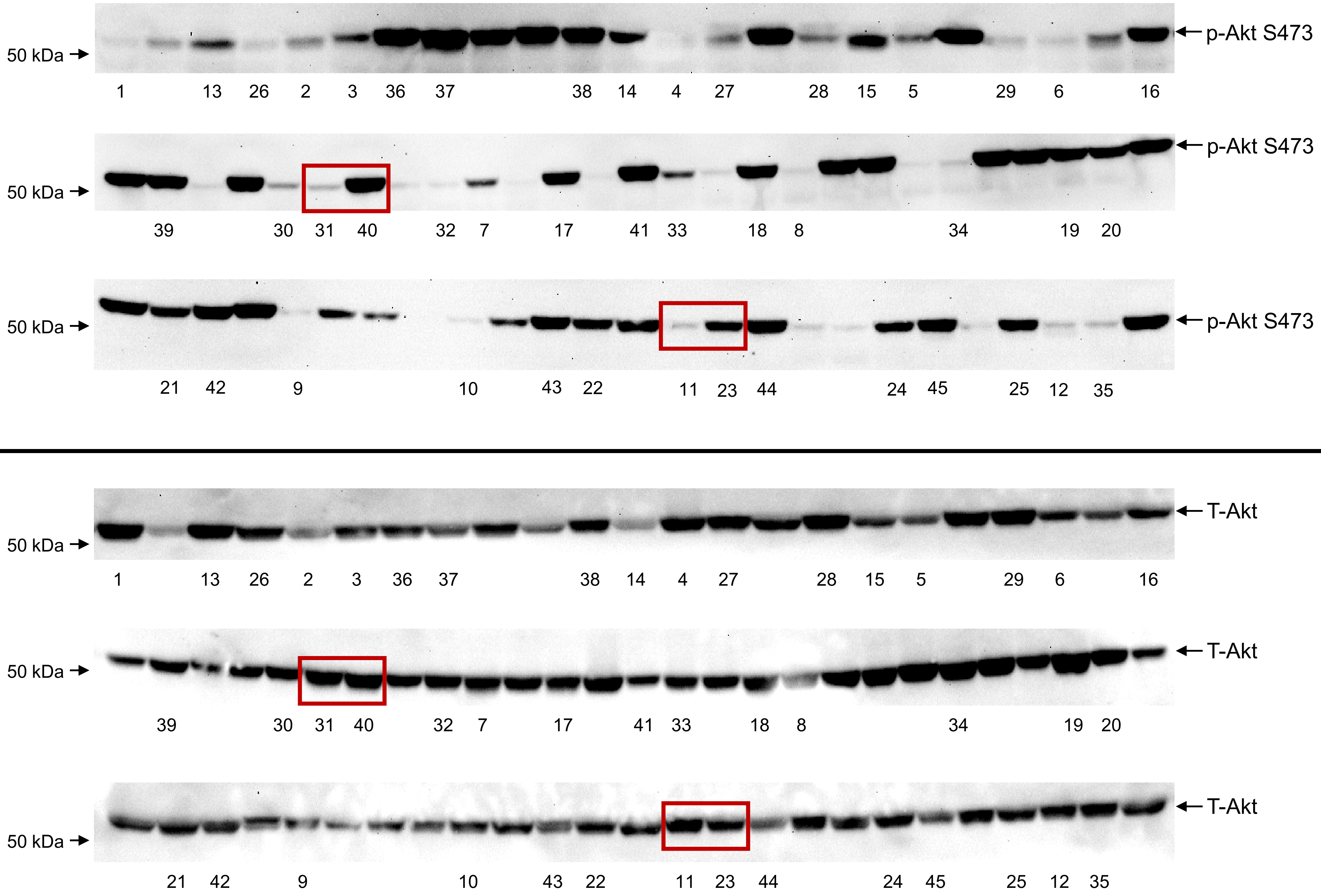
p-Akt S473
50 kDa
1 13 26 2 3 36 37 38 14 4 27 28 15 5 29 6 16
p-Akt S473
50 kDa
39 30 31 40 32 7 17 41 33 18 8 34 19 20
p-Akt S473
50 kDa
21 42 9 10 43 22 11 23 44 24 45 25 12 35
T-Akt
50 kDa
1 13 26 2 3 36 37 38 14 4 27 28 15 5 29 6 16
T-Akt
50 kDa
39 30 31 40 32 7 17 41 33 18 8 34 19 20
T-Akt
50 kDa
21 42 9 10 43 22 11 23 44 24 45 25 12 35

Fig. S2 B

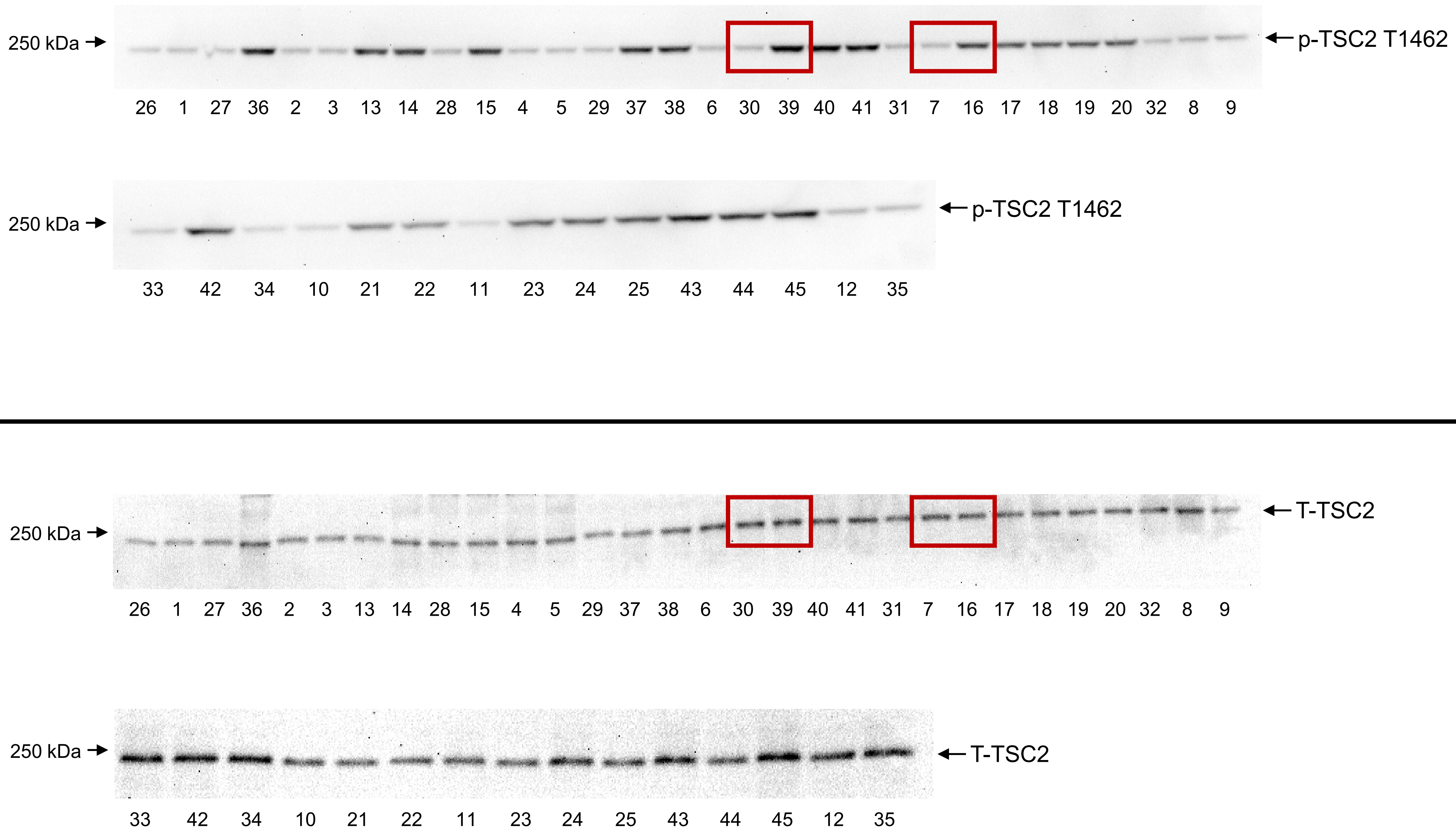
250 kDa
p-TSC2 T1462
26 1 27 36 2 3 13 14 28 15 4 5 29 37 38 6 30 39 40 41 31 7 16 17 18 19 20 32 8 9
250 kDa
p-TSC2 T1462
33 42 34 10 21 22 11 23 24 25 43 44 45 12 35
250 kDa
T-TSC2
26 1 27 36 2 3 13 14 28 15 4 5 29 37 38 6 30 39 40 41 31 7 16 17 18 19 20 32 8 9
250 kDa
T-TSC2
33 42 34 10 21 22 11 23 24 25 43 44 45 12 35

Fig. S2 C

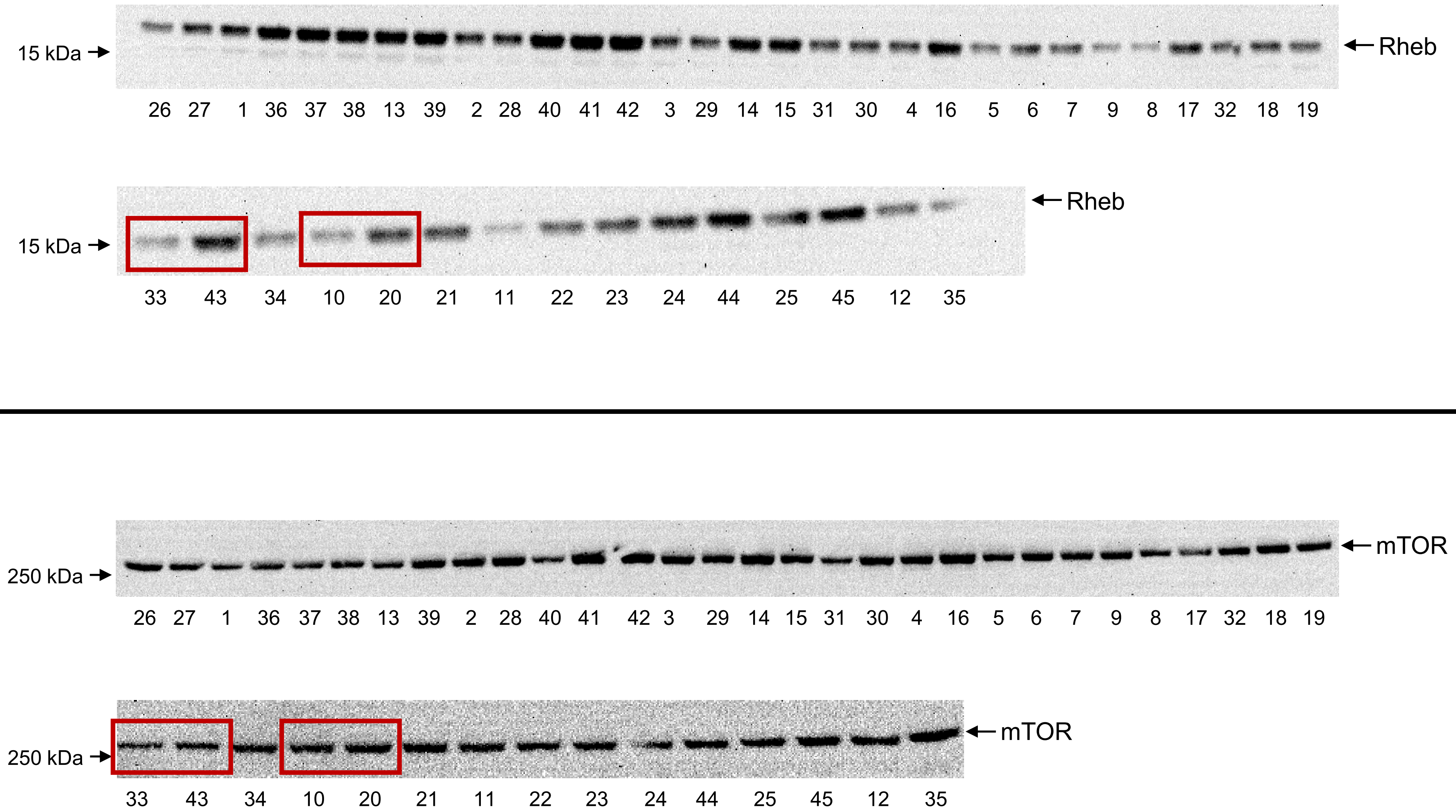

15 kDa
Rheb
26 27 1 36 37 38 13 39 2 28 40 41 42 3 29 14 15 31 30 4 16 5 6 7 9 8 17 32 18 19
15 kDa
Rheb
33 43 34 10 20 21 11 22 23 24 44 25 45 12 35
250 kDa
mTOR
26 27 1 36 37 38 13 39 2 28 40 41 42 3 29 14 15 31 30 4 16 5 6 7 9 8 17 32 18 19
250 kDa
mTOR
33 43 34 10 20 21 11 22 23 24 44 25 45 12 35

Fig. S2 D

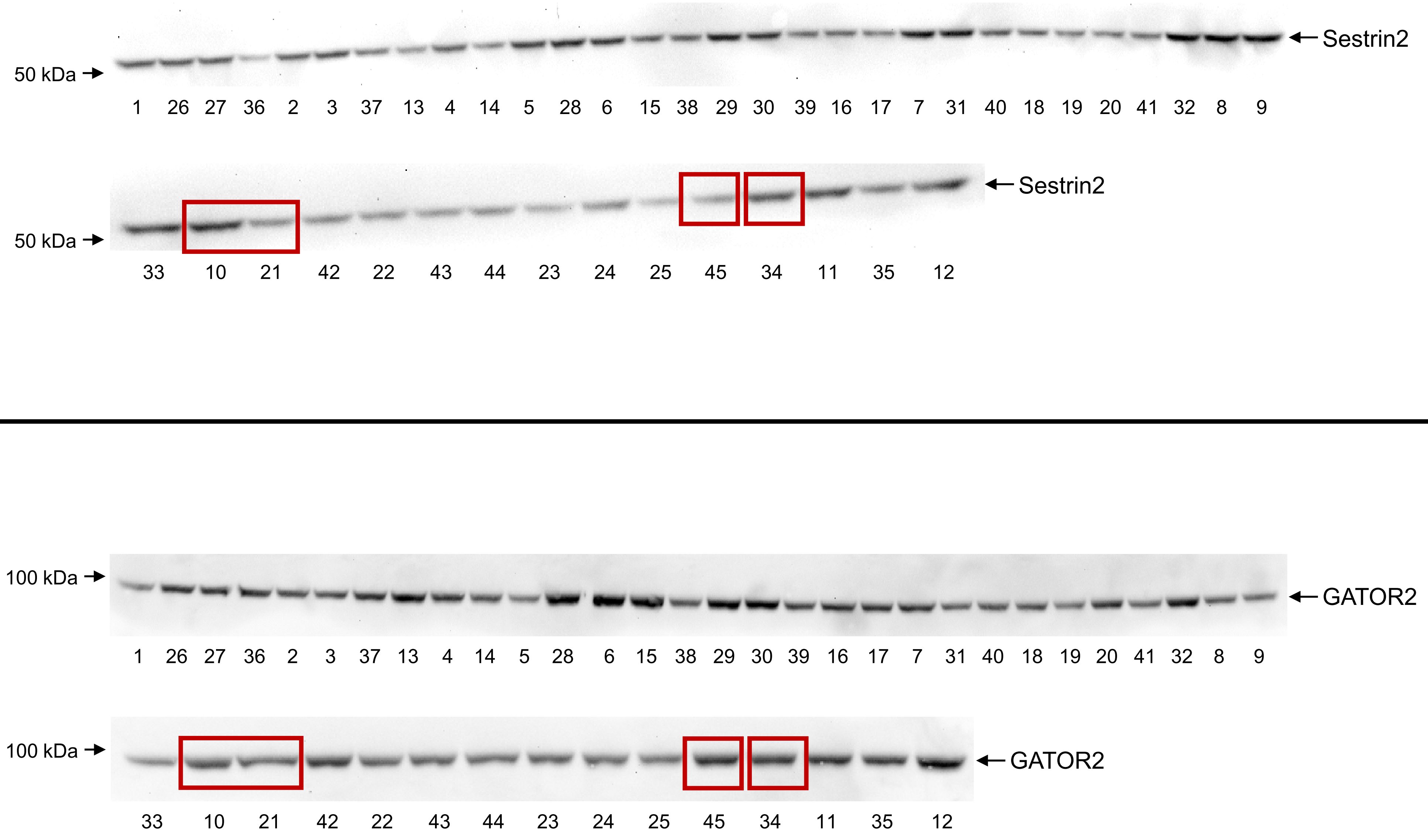
Sestrin2
50 kDa
1 26 27 36 2 3 37 13 4 14 5 28 6 15 38 29 30 39 16 17 7 31 40 18 19 20 41 32 8 9
Sestrin2
50 kDa
33 10 21 42 22 43 44 23 24 25 45 34 11 35 12
100 kDa
GATOR2
1 26 27 36 2 3 37 13 4 14 5 28 6 15 38 29 30 39 16 17 7 31 40 18 19 20 41 32 8 9
100 kDa
GATOR2
33 10 21 42 22 43 44 23 24 25 45 34 11 35 12

Fig. S2 E

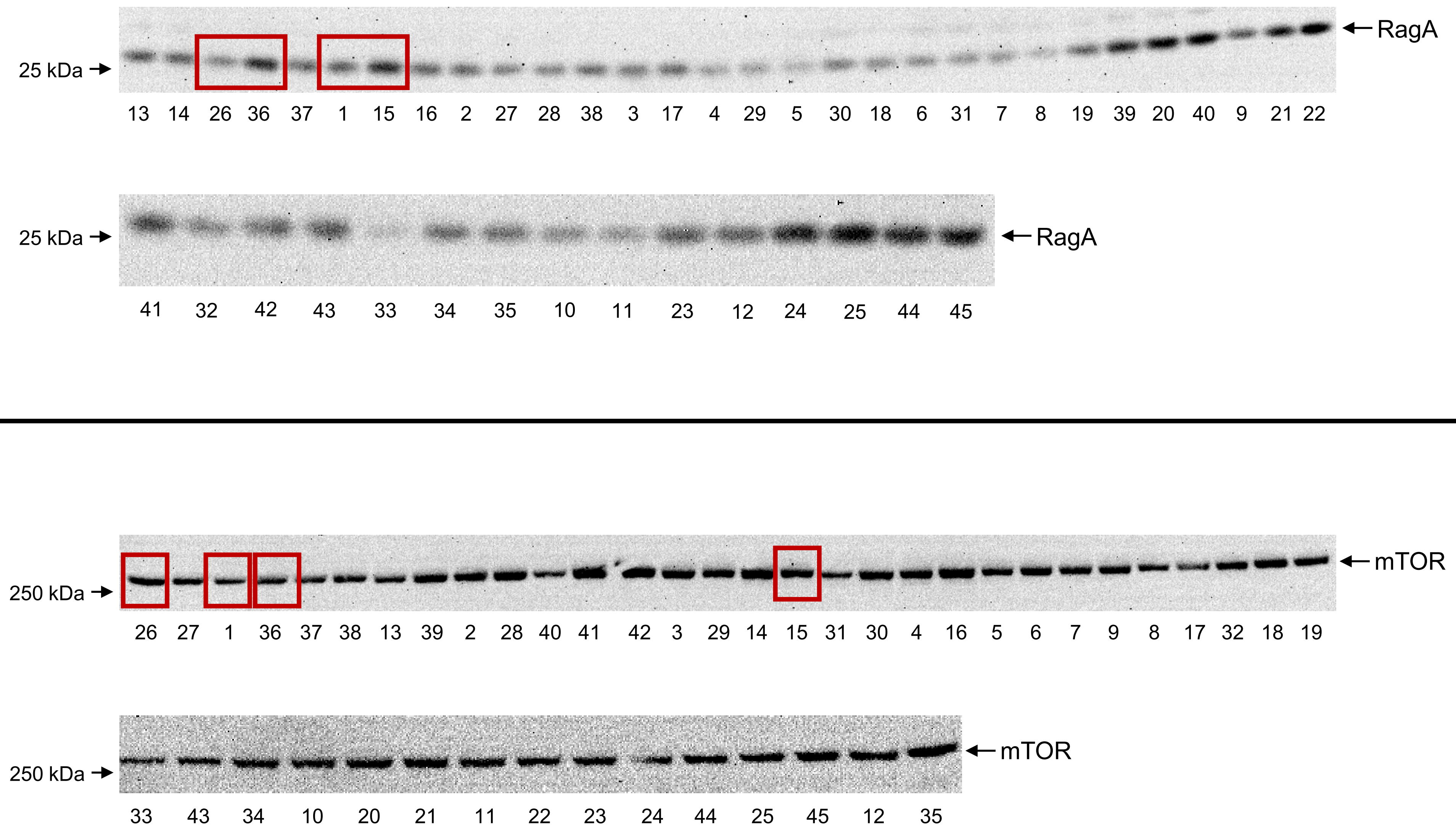
RagA
25 kDa
13 14 26 36 37 1 15 16 2 27 28 38 3 17 4 29 5 30 18 6 31 7 8 19 39 20 40 9 21 22
RagA
25 kDa
41 32 42 43 33 34 35 10 11 23 12 24 25 44 45
mTOR
250 kDa
26 27 1 36 37 38 13 39 2 28 40 41 42 3 29 14 15 31 30 4 16 5 6 7 9 8 17 32 18 19
mTOR
250 kDa
33 43 34 10 20 21 11 22 23 24 44 25 45 12 35

Fig. S2 F

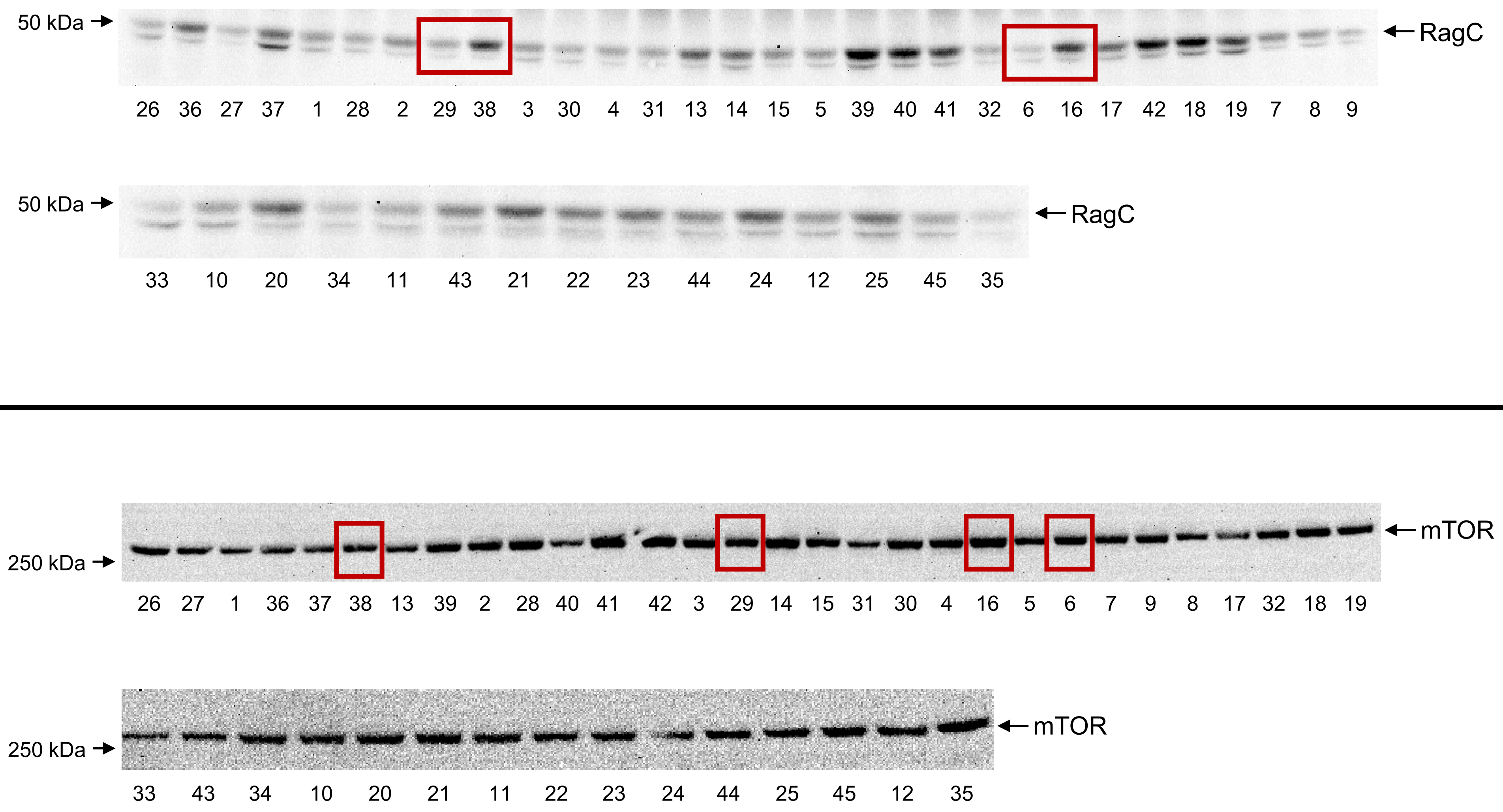
50 kDa
RagC
26 36 27 37 1 28 2 29 38 3 30 4 31 13 14 15 5 39 40 41 32 6 16 17 42 18 19 7 8 9
50 kDa
RagC
33 10 20 34 11 43 21 22 23 44 24 12 25 45 35
mTOR
250 kDa
26 27 1 36 37 38 13 39 2 28 40 41 42 3 29 14 15 31 30 4 16 5 6 7 9 8 17 32 18 19
mTOR
250 kDa
33 43 34 10 20 21 11 22 23 24 44 25 45 12 35

Fig. S3 A

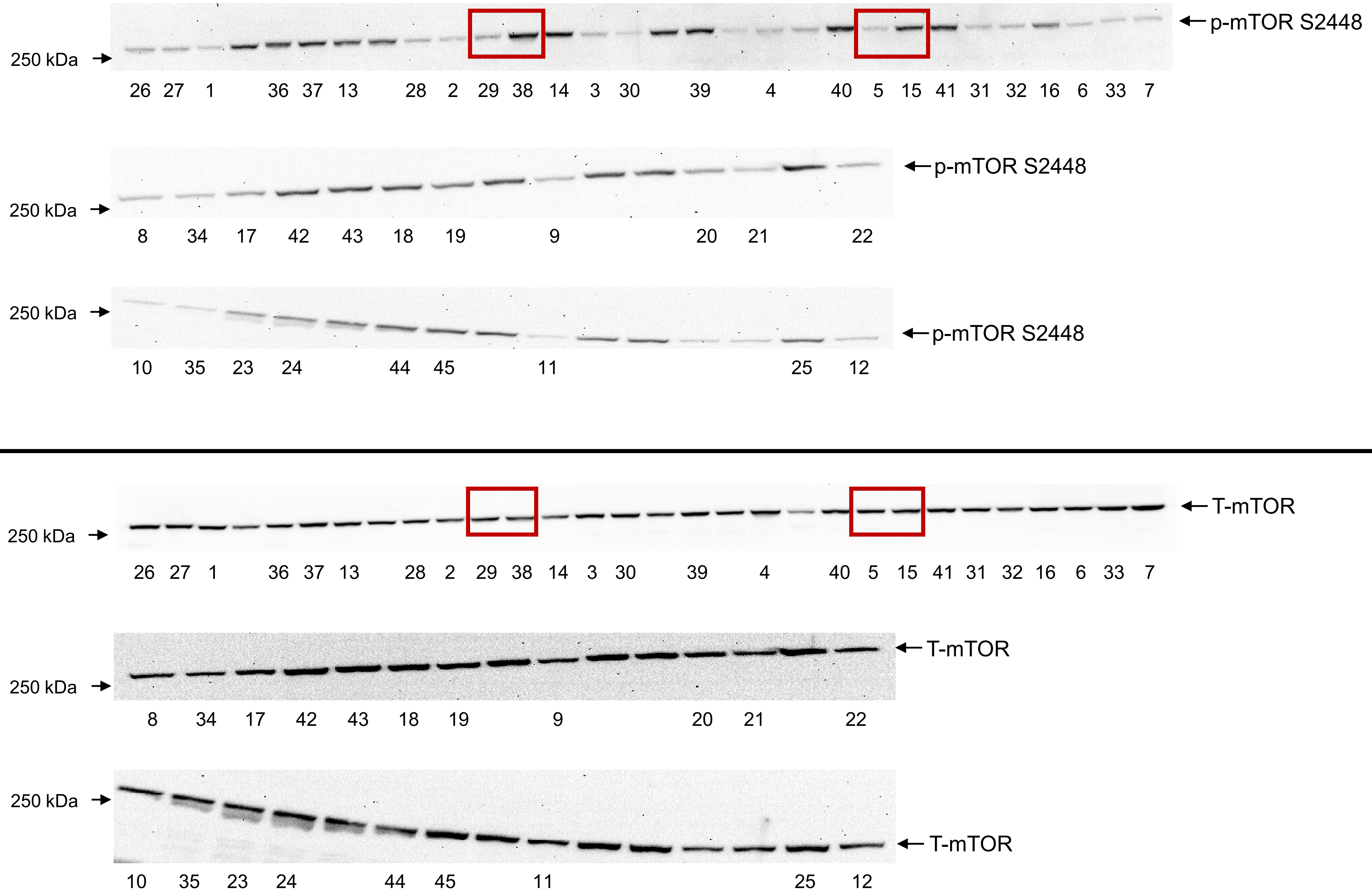
p-mTOR S2448
250 kDa
26 27 1 36 37 13 28 2 29 38 14 3 30 39 4 40 5 15 41 31 32 16 6 33 7
p-mTOR S2448
250 kDa
8 34 17 42 43 18 19 9 20 21 22
250 kDa
p-mTOR S2448
10 35 23 24 44 45 11 25 12
T-mTOR
250 kDa
26 27 1 36 37 13 28 2 29 38 14 3 30 39 4 40 5 15 41 31 32 16 6 33 7
T-mTOR
250 kDa
8 34 17 42 43 18 19 9 20 21 22
250 kDa
T-mTOR
10 35 23 24 44 45 11 25 12

Fig. S3 B

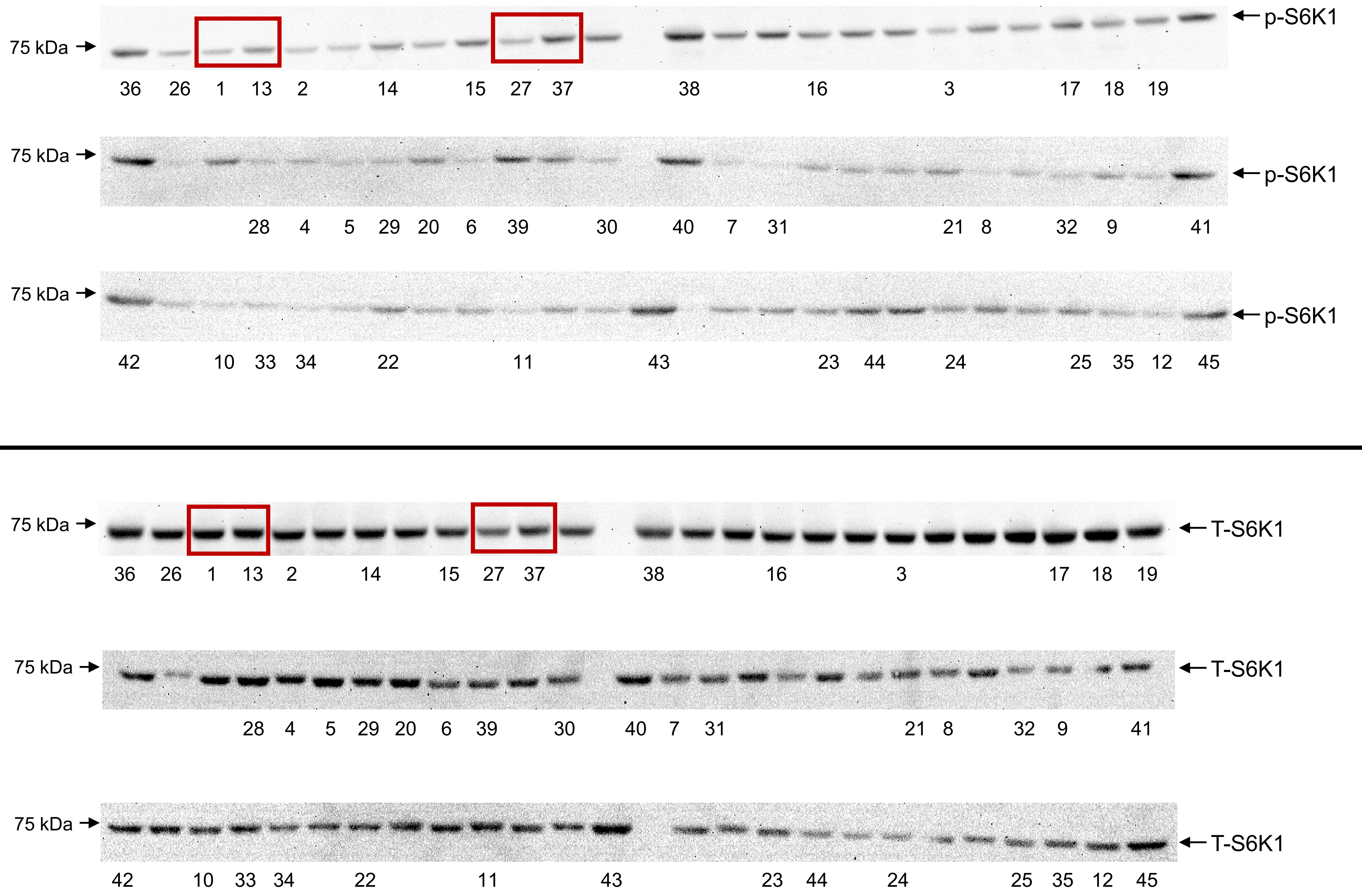

75 kDa
p-S6K1
36 26 1 13 2 14 15 27 37 38 16 3 17 18 19
75 kDa
p-S6K1
28 4 5 29 20 6 39 30 40 7 31 21 8 32 9 41
75 kDa
p-S6K1
42 10 33 34 22 11 43 23 44 24 25 35 12 45
75 kDa
T-S6K1
36 26 1 13 2 14 15 27 37 38 16 3 17 18 19
75 kDa
T-S6K1
28 4 5 29 20 6 39 30 40 7 31 21 8 32 9 41
75 kDa
T-S6K1
42 10 33 34 22 11 43 23 44 24 25 35 12 45

Fig. S3 C

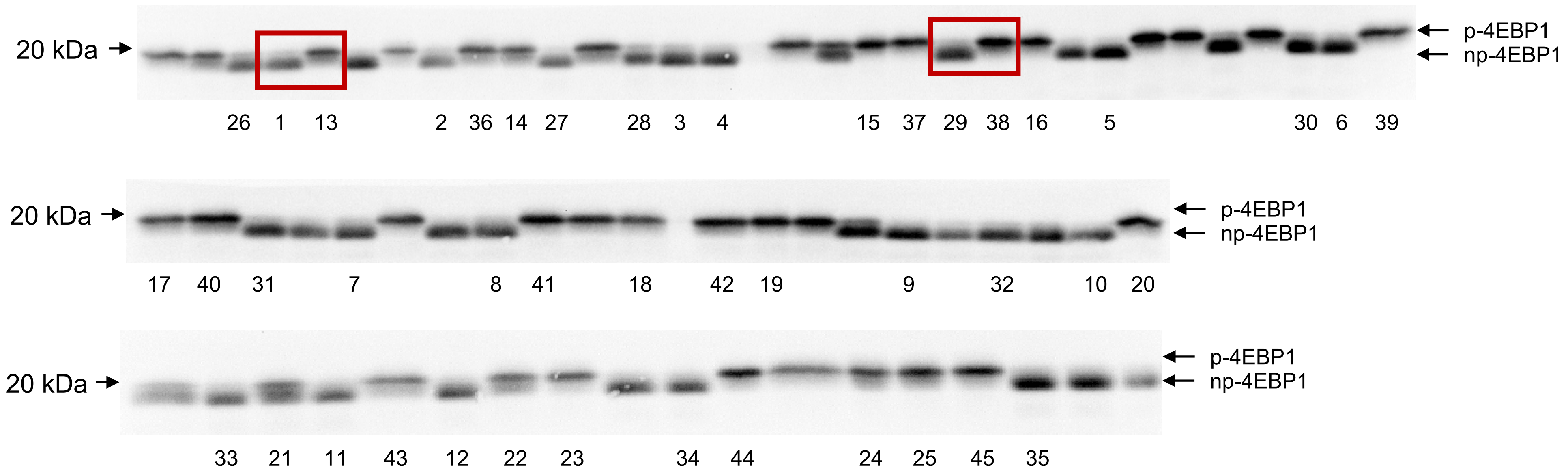
20 kDa
p-4EBP1
np-4EBP1
26 1 13 2 36 14 27 28 3 4 15 37 29 38 16 5 30 6 39
20 kDa
p-4EBP1
np-4EBP1
17 40 31 7 8 41 18 42 19 9 32 10 20
20 kDa
p-4EBP1
np-4EBP1
33 21 11 43 12 22 23 34 44 24 25 45 35

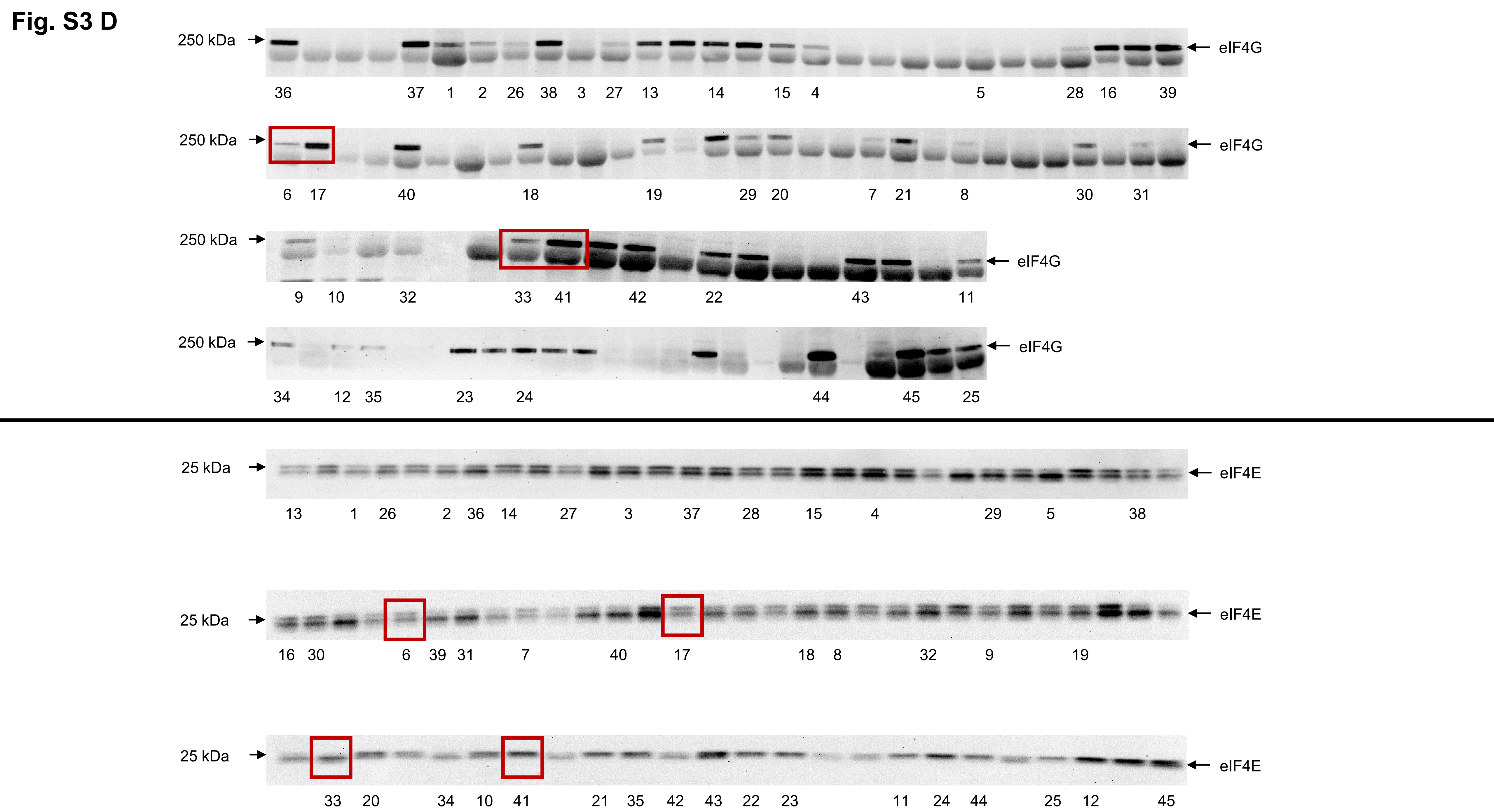
Fig. S3 D
250 kDa
eIF4G
36 37 1 2 26 38 3 27 13 14 15 4 5 28 16 39
250 kDa
eIF4G
6 17 40 18 19 29 20 7 21 8 30 31
250 kDa
eIF4G
9 10 32 33 41 42 22 43 11
250 kDa
eIF4G
34 12 35 23 24 44 45 25
25 kDa
eIF4E
13 1 26 2 36 14 27 3 37 28 15 4 29 5 38
25 kDa
eIF4E
16 30 6 39 31 7 40 17 18 8 32 9 19
25 kDa
eIF4E
33 20 34 10 41 21 35 42 43 22 23 11 24 44 25 12 45

Fig. S3 E

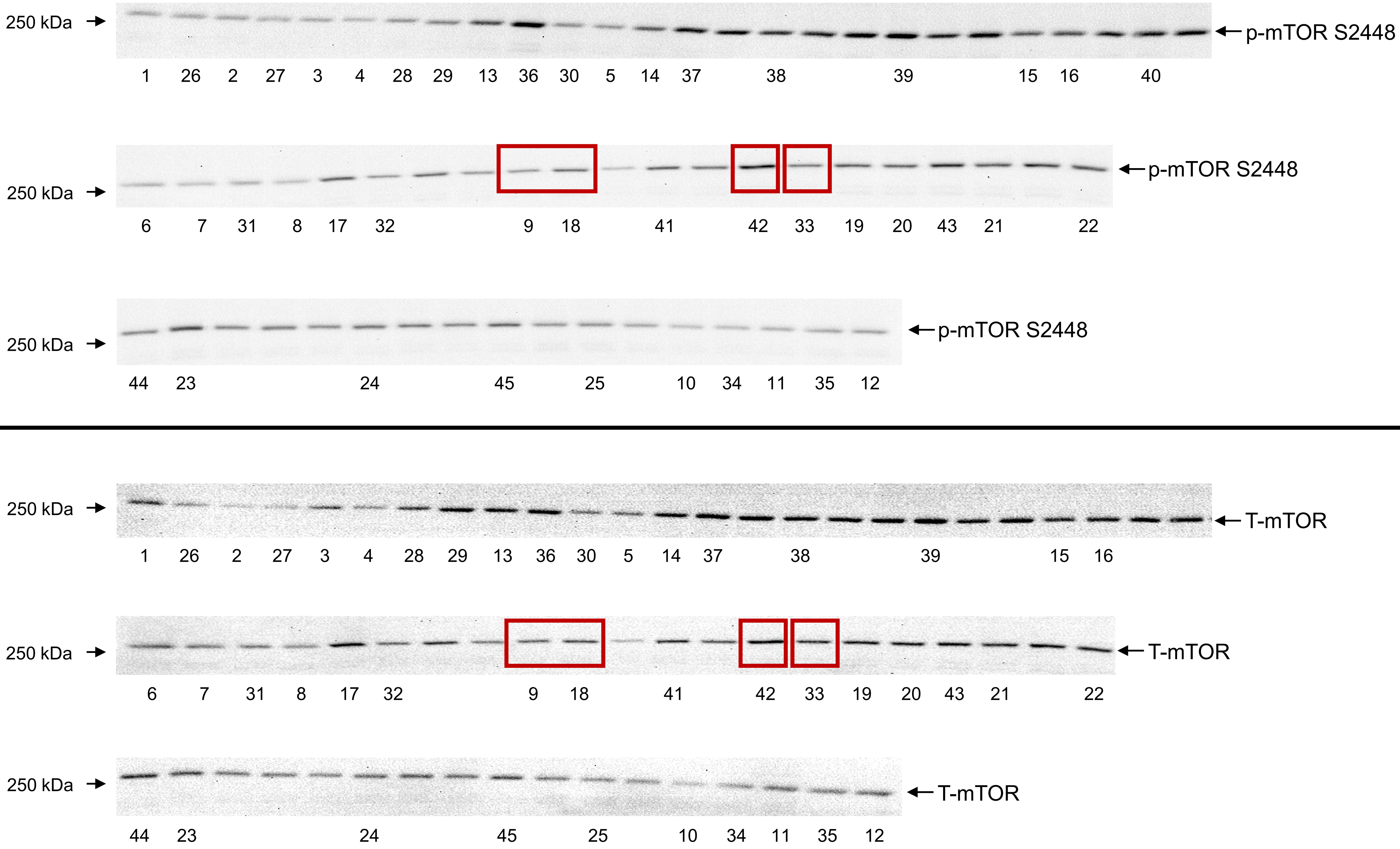
250 kDa
p-mTOR S2448
1 26 2 27 3 4 28 29 13 36 30 5 14 37 38 39 15 16 40
250 kDa
p-mTOR S2448
6 7 31 8 17 32 9 18 41 42 33 19 20 43 21 22
250 kDa
p-mTOR S2448
44 23 24 45 25 10 34 11 35 12
250 kDa
T-mTOR
1 26 2 27 3 4 28 29 13 36 30 5 14 37 38 39 15 16
250 kDa
T-mTOR
6 7 31 8 17 32 9 18 41 42 33 19 20 43 21 22
250 kDa
T-mTOR
44 23 24 45 25 10 34 11 35 12

Fig. S3 F

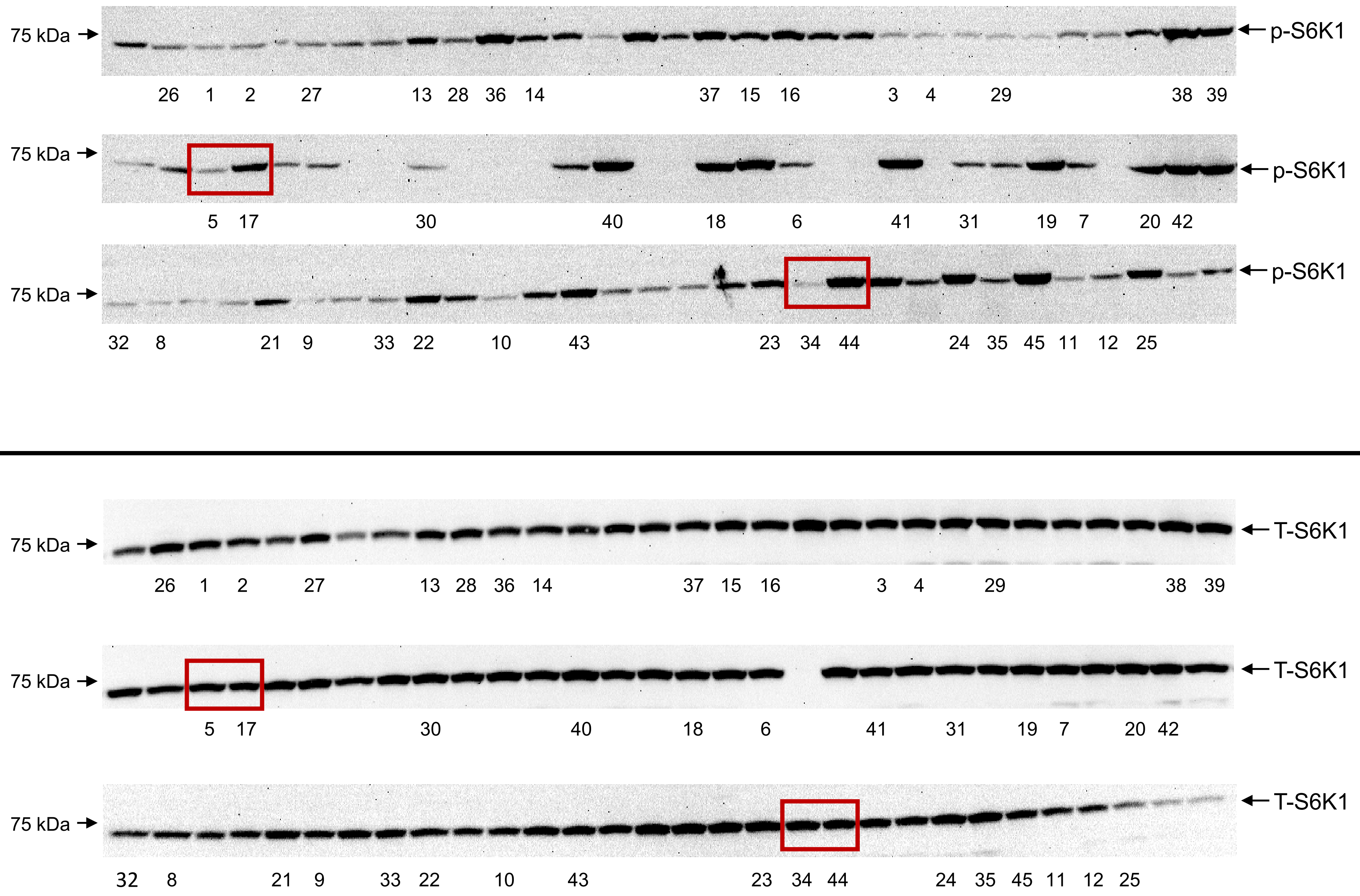
75 kDa
p-S6K1
26 1 2 27 13 28 36 14 37 15 16 3 4 29 38 39
75 kDa
p-S6K1
5 17 30 40 18 6 41 31 19 7 20 42
75 kDa
p-S6K1
32 8 21 9 33 22 10 43 23 34 44 24 35 45 11 12 25
75 kDa
T-S6K1
26 1 2 27 13 28 36 14 37 15 16 3 4 29 38 39
75 kDa
T-S6K1
5 17 30 40 18 6 41 31 19 7 20 42
75 kDa
T-S6K1
32 8 21 9 33 22 10 43 23 34 44 24 35 45 11 12 25

Fig. S3 G

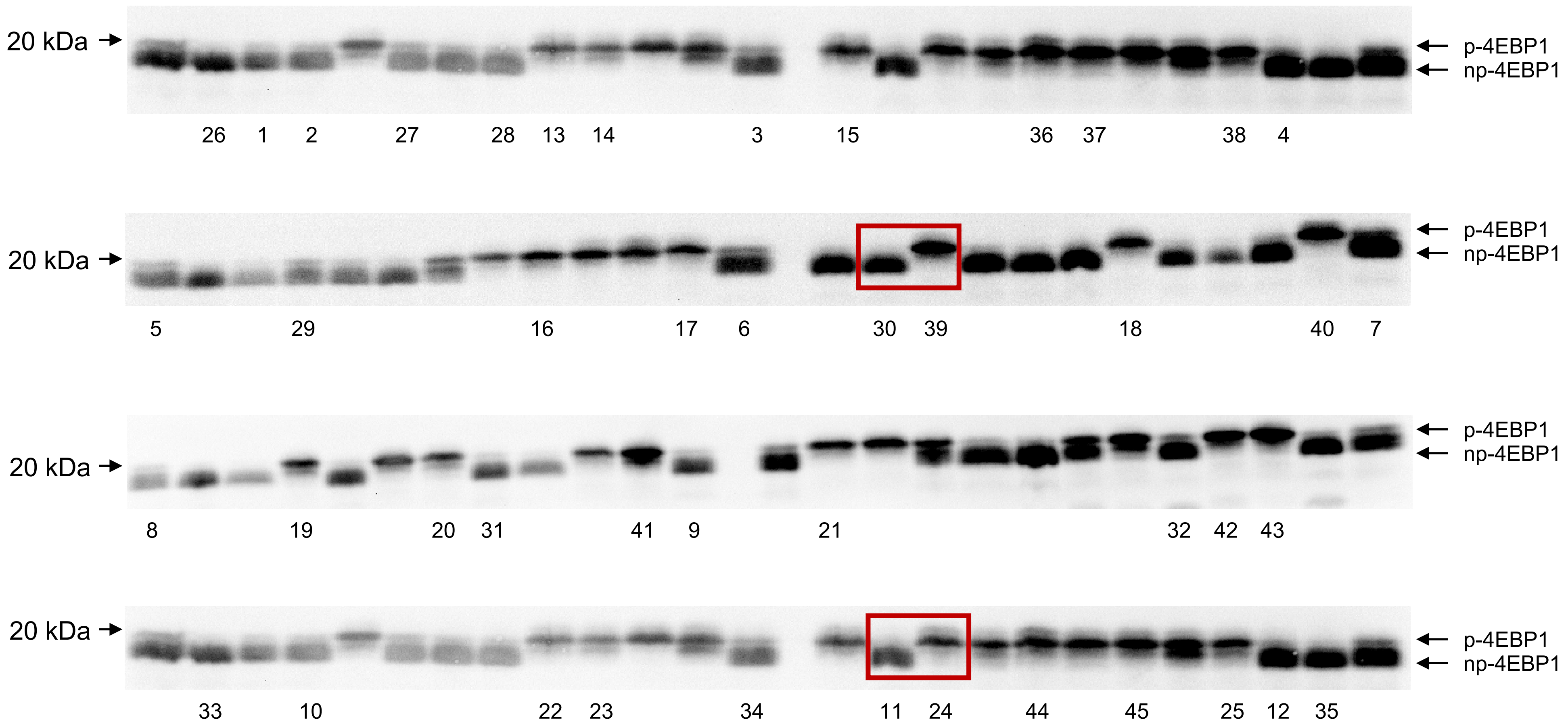
20 kDa
p-4EBP1
np-4EBP1
26 1 2 27 28 13 14 3 15 36 37 38 4
20 kDa
p-4EBP1
np-4EBP1
5 29 16 17 6 30 39 18 40 7
20 kDa
p-4EBP1
np-4EBP1
8 19 20 31 41 9 21 32 42 43
20 kDa
p-4EBP1
np-4EBP1
33 10 22 23 34 11 24 44 45 25 12 35

Fig. S3 H

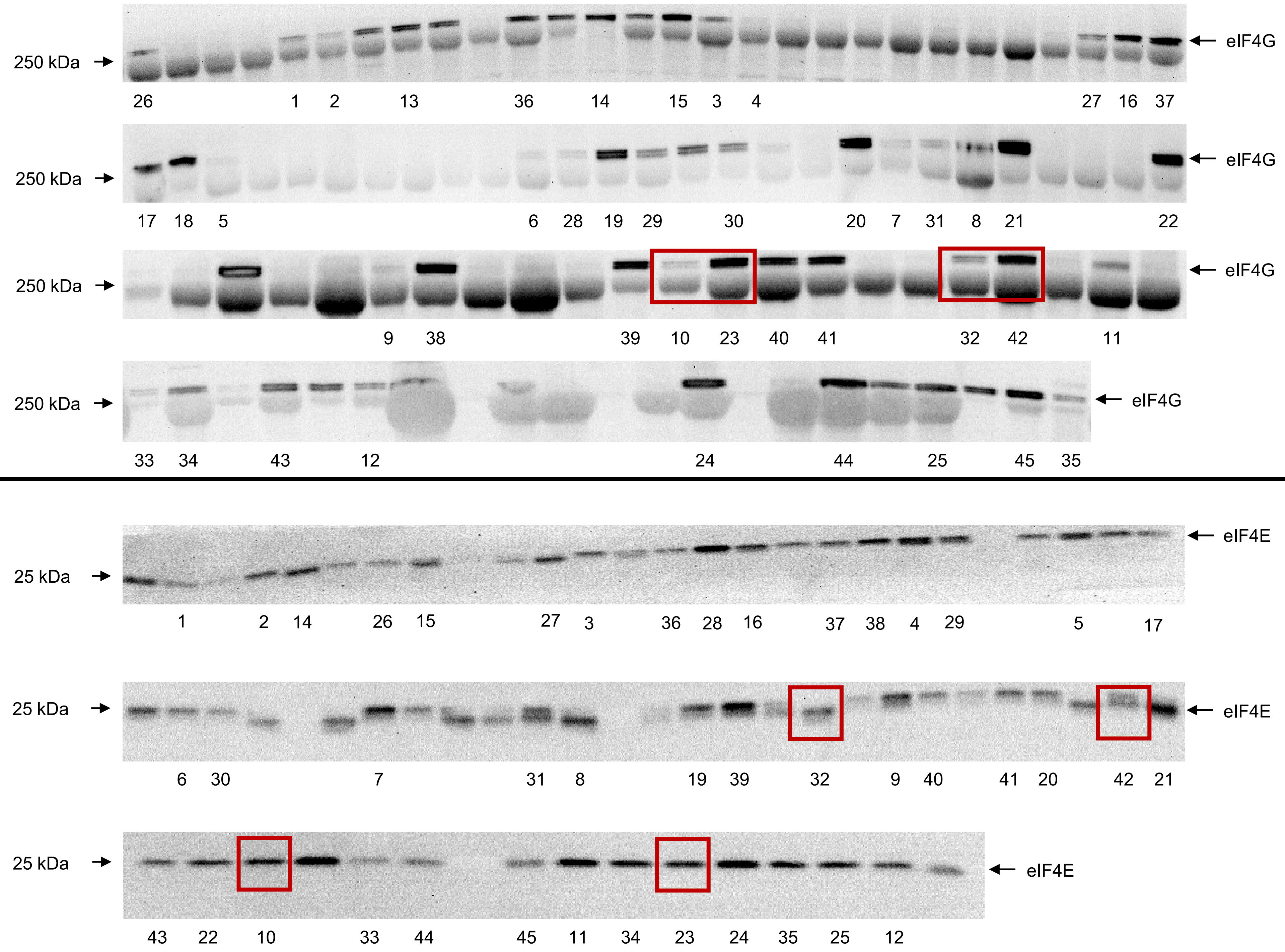
250 kDa
eIF4G
26 1 2 13 36 14 15 3 4 27 16 37
250 kDa
eIF4G
17 18 5 6 28 19 29 30 20 7 31 8 21 22
250 kDa
eIF4G
9 38 39 10 23 40 41 32 42 11
250 kDa
eIF4G
33 34 43 12 24 44 25 45 35
25 kDa
eIF4E
1 2 14 26 15 27 3 36 28 16 37 38 4 29 5 17
25 kDa
eIF4E
6 30 7 31 8 19 39 32 9 40 41 20 42 21
25 kDa
eIF4E
43 22 10 33 44 45 11 34 23 24 35 25 12

Fig. S4 A

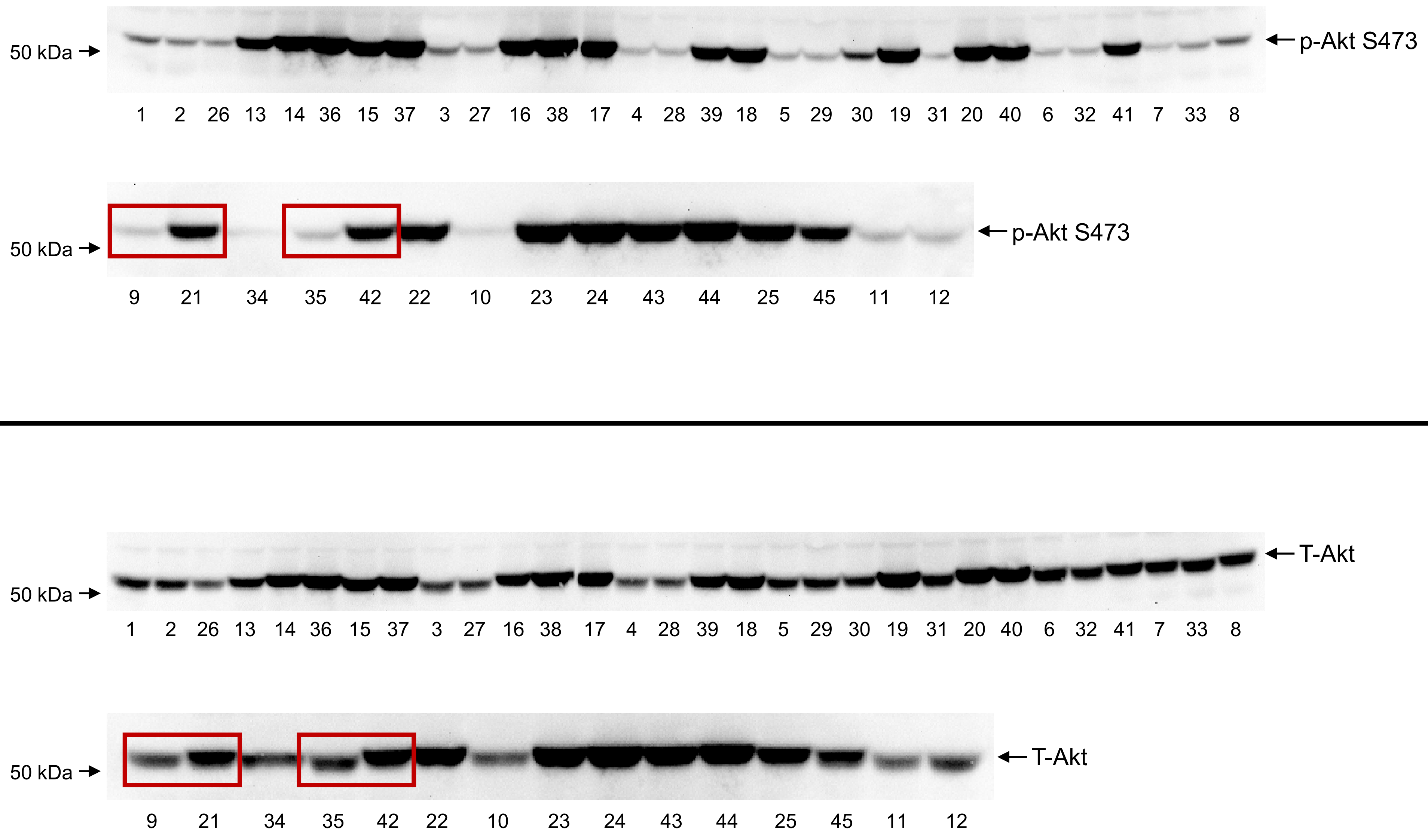
50 kDa
p-Akt S473
1 2 26 13 14 36 15 37 3 27 16 38 17 4 28 39 18 5 29 30 19 31 20 40 6 32 41 7 33 8
50 kDa
p-Akt S473
9 21 34 35 42 22 10 23 24 43 44 25 45 11 12
50 kDa
T-Akt
1 2 26 13 14 36 15 37 3 27 16 38 17 4 28 39 18 5 29 30 19 31 20 40 6 32 41 7 33 8
50 kDa
T-Akt
9 21 34 35 42 22 10 23 24 43 44 25 45 11 12

Fig. S4 B

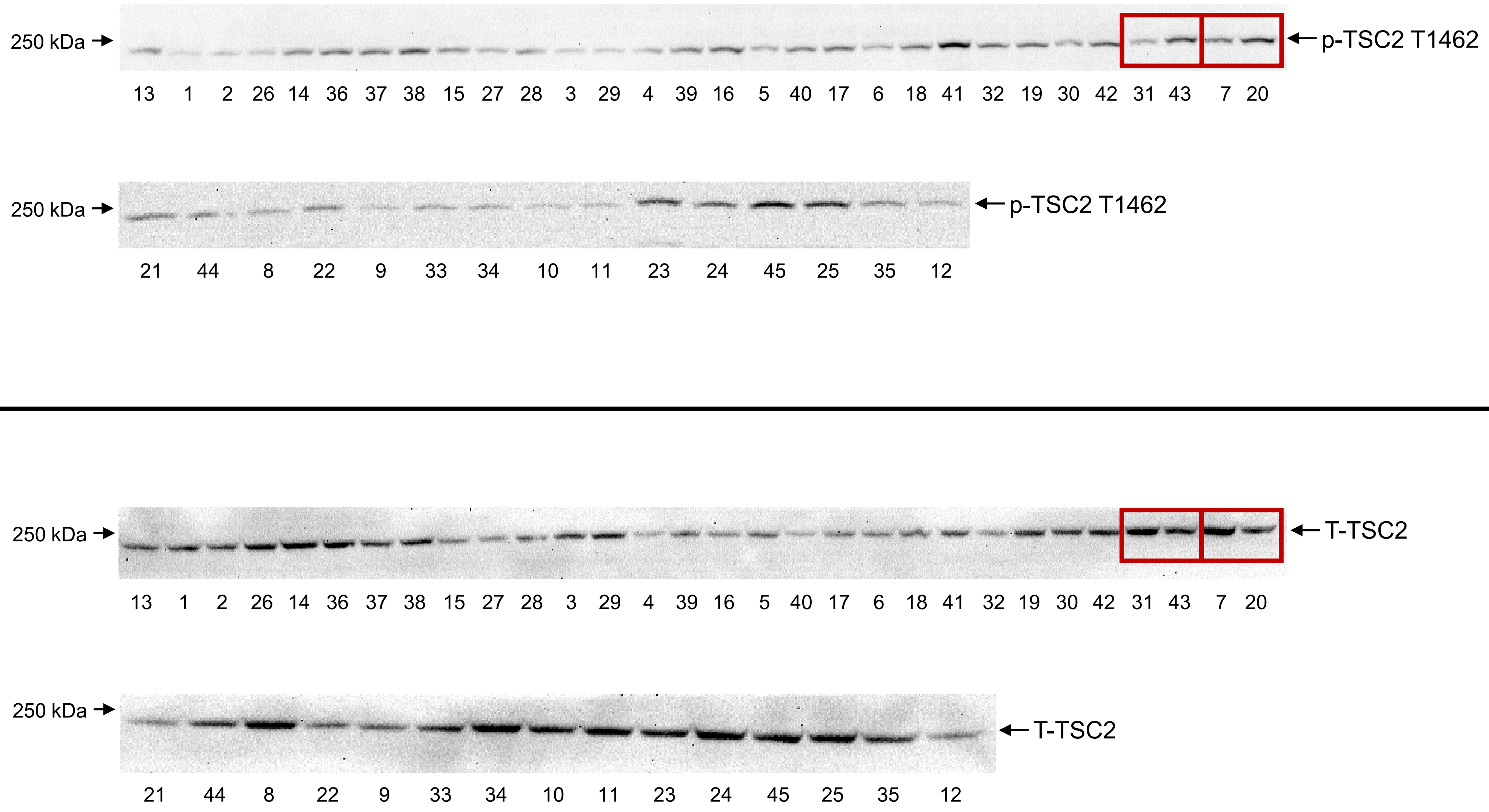
250 kDa
p-TSC2 T1462
13 1 2 26 14 36 37 38 15 27 28 3 29 4 39 16 5 40 17 6 18 41 32 19 30 42 31 43 7 20
250 kDa
p-TSC2 T1462
21 44 8 22 9 33 34 10 11 23 24 45 25 35 12
250 kDa
T-TSC2
13 1 2 26 14 36 37 38 15 27 28 3 29 4 39 16 5 40 17 6 18 41 32 19 30 42 31 43 7 20
250 kDa
T-TSC2
21 44 8 22 9 33 34 10 11 23 24 45 25 35 12

Fig. S4 C

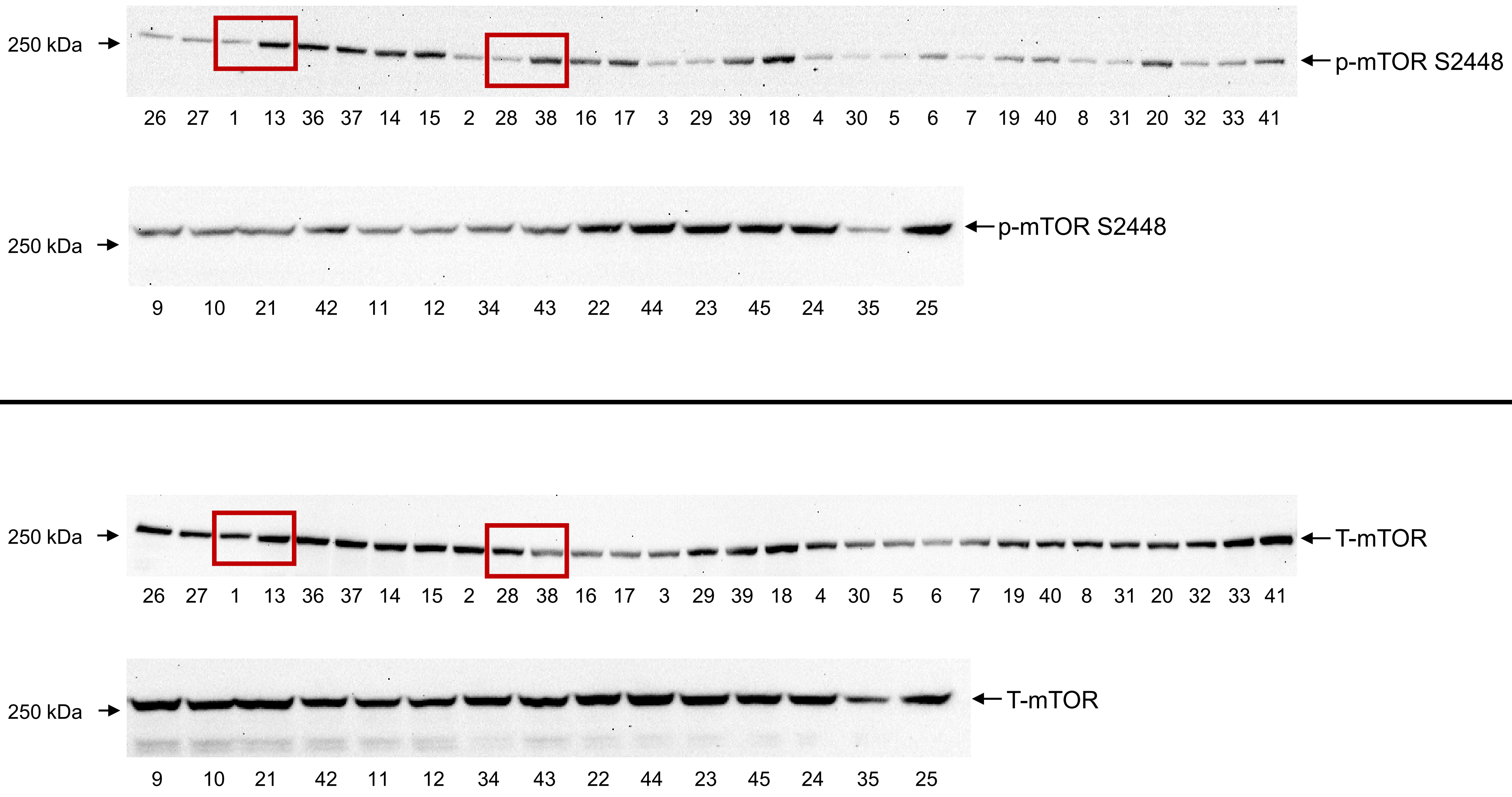
250 kDa
p-mTOR S2448
26 27 1 13 36 37 14 15 2 28 38 16 17 3 29 39 18 4 30 5 6 7 19 40 8 31 20 32 33 41
250 kDa
p-mTOR S2448
9 10 21 42 11 12 34 43 22 44 23 45 24 35 25
250 kDa
T-mTOR
26 27 1 13 36 37 14 15 2 28 38 16 17 3 29 39 18 4 30 5 6 7 19 40 8 31 20 32 33 41
250 kDa
T-mTOR
9 10 21 42 11 12 34 43 22 44 23 45 24 35 25

Fig. S4 D

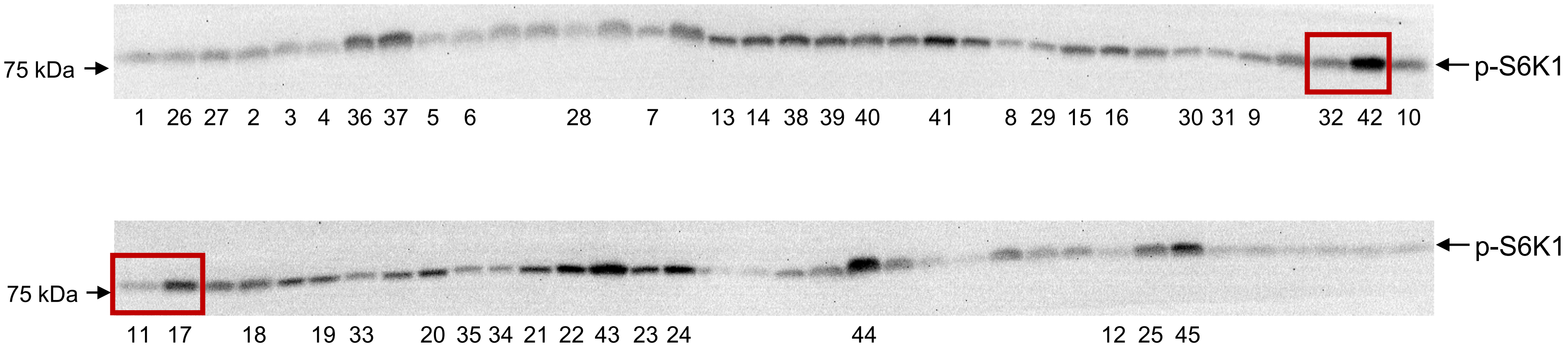
75 kDa
p-S6K1
1 26 27 2 3 4 36 37 5 6 28 7 13 14 38 39 40 41 8 29 15 16 30 31 9 32 42 10
75 kDa
p-S6K1
11 17 18 19 33 20 35 34 21 22 43 23 24 44 12 25 45

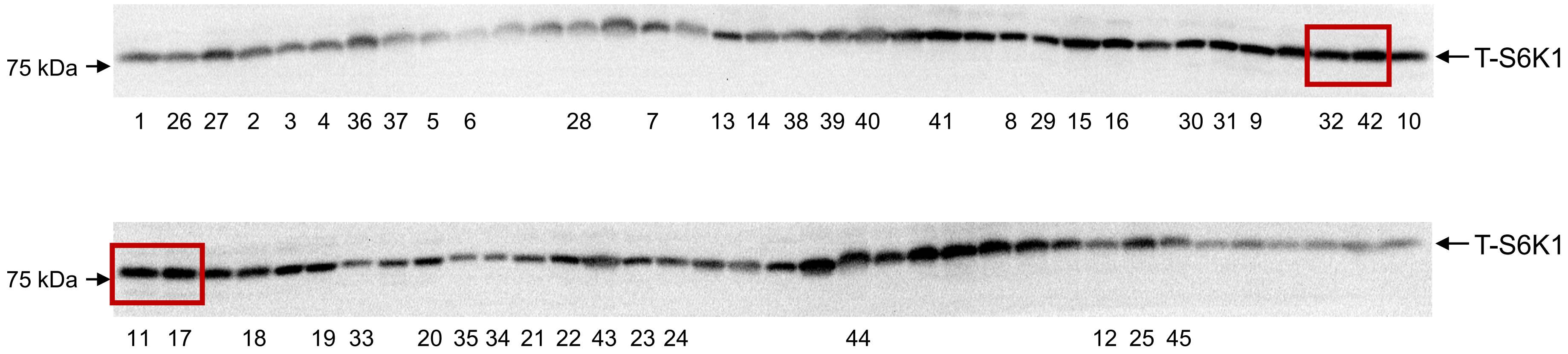
75 kDa
T-S6K1
1 26 27 2 3 4 36 37 5 6 28 7 13 14 38 39 40 41 8 29 15 16 30 31 9 32 42 10
75 kDa
T-S6K1
11 17 18 19 33 20 35 34 21 22 43 23 24 44 12 25 45

Fig. S4 E

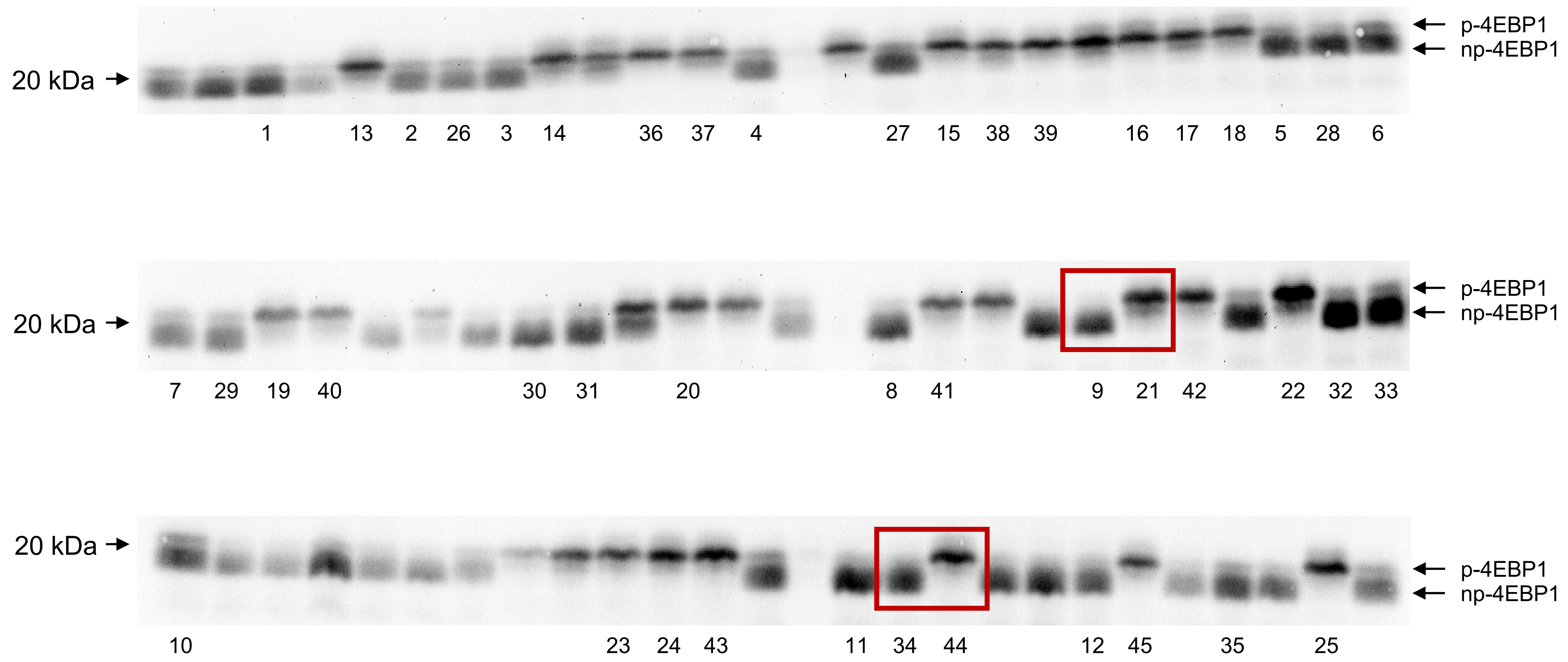
20 kDa
p-4EBP1
np-4EBP1
1 13 2 26 3 14 36 37 4 27 15 38 39 16 17 18 5 28 6
20 kDa
p-4EBP1
np-4EBP1
7 29 19 40 30 31 20 8 41 9 21 42 22 32 33
20 kDa
p-4EBP1
np-4EBP1
10 23 24 43 11 34 44 12 45 35 25

Fig. S4 F

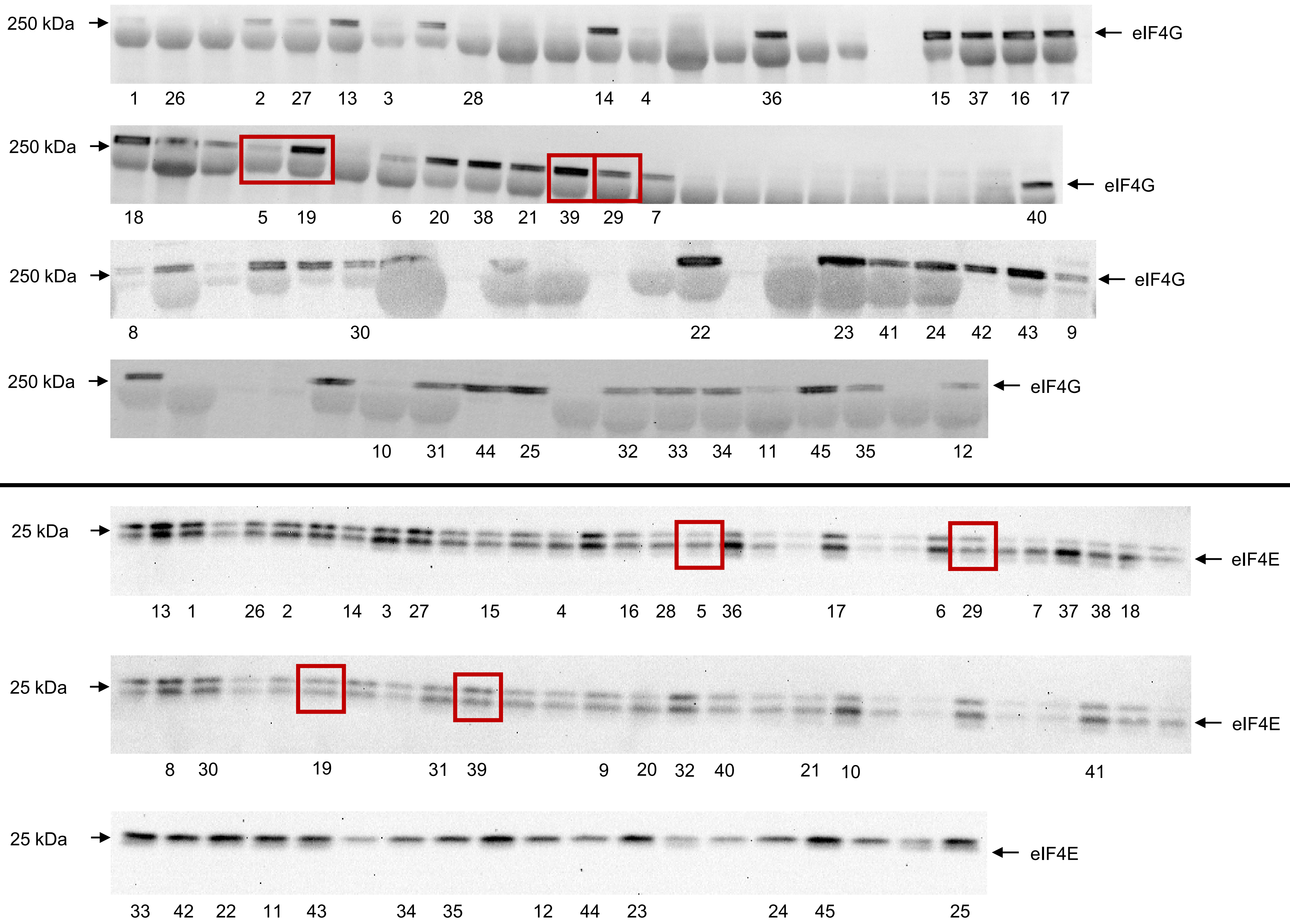

250 kDa
eIF4G
1 26 2 27 13 3 28 14 4 36 15 37 16 17
250 kDa
eIF4G
18 5 19 6 20 38 21 39 29 7 40
250 kDa
eIF4G
8 30 22 23 41 24 42 43 9
250 kDa
eIF4G
10 31 44 25 32 33 34 11 45 35 12
25 kDa
eIF4E
13 1 26 2 14 3 27 15 4 16 28 5 36 17 6 29 7 37 38 18
25 kDa
eIF4E
8 30 19 31 39 9 20 32 40 21 10 41
25 kDa
eIF4E
33 42 22 11 43 34 35 12 44 23 24 45 25
